# Alcohol and Health Outcomes: An Umbrella Review of Meta-Analyses Base on Prospective Cohort Studies

**DOI:** 10.3389/fpubh.2022.859947

**Published:** 2022-05-04

**Authors:** Lixian Zhong, Weiwei Chen, Tonghua Wang, Qiuting Zeng, Leizhen Lai, Junlong Lai, Junqin Lin, Shaohui Tang

**Affiliations:** ^1^Department of Gastroenterology, The First Affiliated Hospital, Jinan University, Guangzhou, China; ^2^Department of Gastroenterology, The First People's Hospital of Zunyi (The Third Affiliated Hospital of Zunyi Medical University), Zunyi, China; ^3^Department of Gastroenterology, Affiliated Hospital of Youjiang Medical University for Nationalities, Baise, China

**Keywords:** alcohol, health outcomes, umbrella review, meta-analysis, epidemiologic evidence

## Abstract

An umbrella review of meta-analyses was performed to summarize the evidence of associations between alcohol consumption and health outcomes and to assess its credibility. Meta-analyses of prospective cohort studies reporting the associations of alcohol consumption with health outcomes were identified. We recalculated the random-effects summary effect size and 95% confidence interval, heterogeneity, and small-study effect for each meta-analysis and graded the evidence. Fifty-nine publications reporting 224 meta-analyses of prospective cohort studies with 140 unique health outcomes were included, in which there were 49 beneficial associations and 25 harmful associations with nominally statistically significant summary results. But quality of evidence was rated high only for seven beneficial associations (renal cell carcinoma risk, dementia risk, colorectal cancer mortality, and all-cause mortality in patients with hypertension for low alcohol consumption; renal cell carcinoma risk, cardiovascular disease (CVD) risk in patients with hypertension and all-cause mortality in patients with hypertension for moderate consumption) and four harmful associations (cutaneous basal cell carcinoma risk for low alcohol consumption; cutaneous basal cell carcinoma risk and cutaneous squamous cell carcinoma risk for moderate alcohol consumption; hemorrhagic stroke risk for high alcohol consumption). In this umbrella review, only 11 health outcomes (5 in low alcohol consumption, 5 in moderate alcohol consumption and 1 in high alcohol consumption) with statistically significant showed high quality of epidemiologic evidence. More robust and larger prospective studies are needed to verify our results.

## Introduction

According to the data from WHO in 2018, about 2.3 billion people worldwide are current drinkers (1). Population surveys indicate that 12 to 14% of adults have current alcohol abuse and 29% had such disorder at some point in their lifetime (2, 3). More than 40 diseases and 2.8 million deaths were fully attributable to alcohol in 2016, which has aroused widespread concern and caused substantial health loss (4, 5). The American Society of Clinical Oncology (6) stated that alcohol is a cause of cancers of the oral cavity, pharynx, larynx, esophagus, liver and breast cancer. However, there has no evidence to assess the relationship between alcohol and other cancers such as endometrial cancer, ovarian cancer, renal cell carcinoma and so on. In recent decades, it is convincing that alcohol consumption had protective effects against cardiovascular disease (7–9), including total cardiovascular disease (CVD), CVD mortality, myocardial infarction (MI), coronary heart disease (CHD), ischemic stroke and heart failure. In the meanwhile, the relationship between nervous system diseases, hematological malignancy and other health outcomes are still unclear. Thus, there have been inconsistent conclusions as to the associations of alcohol consumption with human health outcomes.

Umbrella review is becoming more and more important for overviewing evidence of published systematic reviews and meta-analyses on a specific topic. Only one recent report that indicated the relationship between different types of alcohol and partial health outcomes of meta-analyses of observational studies (10). To our knowledge, there is no existing umbrella review of meta-analyses of prospective cohort studies to capture the breadth of outcomes associated with alcohol consumption and assess the quality of evidence and methodology.

Consequently, we performed this umbrella review of meta-analyses that only including prospective cohort studies to comprehensively assess the methodological quality and investigate the potential bias. More importantly, we evaluated the evidence's breadth, strength, and validity on the associations of alcohol consumption with multiple health outcomes. We believe that our work can provide a more concrete basis for formulation of alcohol consumption guidelines.

## Methods

Our protocol has been registered in PROSPERO (CRD42021228480). We followed the preferred reporting items for systematic reviews and meta-analyses (PRISMA) Guidelines (11).

### Search Strategy

The systematic literature search was conducted in PubMed, Web of Science and EMBASE with no time limit for meta-analyses of prospective observational studies. Search items were “alcohol and meta-analysis,” and no other restrictions were imposed. The literature search was conducted by three authors (Zhong, Chen, and Wang). Disagreements were resolved by consensus.

### Selection Criteria

Meta-analyses were included if they met the following criteria: (1) Meta-analyses included prospective cohort studies; (2) Meta-analyses reported the relationship between alcohol consumption and direct results on human outcome (incidence or mortality of diseases). The protocols, abstracts of the conference, and letters to editors were excluded. We also excluded the study involves animal research and indirect indicators on human health. When several meta-analyses simultaneously reported the same health outcome, we included the one with the largest number of studies.

### Data Extraction

One author (LZ) extracted data separately checked by the other authors (WC and TW). For each included meta-analysis, we extracted the following data: the first author's name and publication year. For each primary study in the included meta-analyses, we extracted the name of the first author, publication year, the number of cases and participants, exposure, relative risk (RR), hazard ratios (HR), odds ratios (OR), 95% confidence intervals. Since the amount of alcohol exposure varies in the primary studies, we uniformly classified alcohol consumption levels into three groups based on the primary studies: low, moderate, and high, which were defined as ethanol intake of **>**0 and ≤ 14.9 g/day (about >0 and <1 drink/day), 15–9.9 g/day (about 1–2.5 drinks/day), ≥30 g/day (about >2.5 drinks/day), respectively (9). The primary prospective studies in each included meta-analysis were retained if they met the following criteria: (1) The primary studies reported the number of cases and participants; (2) Reference groups were non-drinker. For the overlapping meta-analyses, we selected the one with the largest number of cases and participants combined.

### Assessment of Methodological Quality

We used the validated AMSTAR 2 tool (12) to evaluate the methodological quality of each included published meta-analysis of prospective studies. It has been proven to be an effective and reliable tool for assessing the quality of systematic evaluation methodologies. The AMSTAR tool includes 16 items about the conduction of a meta-analysis. No or only one non-critical defect is considered high methodologic quality, and more than one non-critical defect is considered moderate methodologic quality. Only one critical weakness with or without non-critical defects is considered low method quality, and more than one critical weakness with or without critical defects is critically low methodologic quality. Discrepancies between AMSTAR 2 scores were resolved by discussion.

### Evaluation of the Grading of Evidence

We classified evidence from meta-analyses of observational studies with nominally statistically significant summary results into three categories (high, moderate, and weak). We evaluated the strength of epidemiologic evidence with the following criteria (13–17): (1) precision of the estimate [*P* < 0.001 (18, 19), a threshold associated with significantly fewer false-positive results, and more than 1,000 cases of the disease]; (2) consistency of results (I2 <50%; Cochran Q test, *P* > 0.10); (3) no evidence of small-study effects (*P* > 0.10). If all these criteria were satisfied, the strength of the epidemiologic evidence was rated as high. If a maximum of 1 criterion was not satisfied and *P* < 0.001 was found, the strength of the epidemiologic evidence was rated as moderate. If it is all other cases (*P* < 0.05), the strength of the epidemiologic evidence was considered weak.

### Data Analysis

According to the above information, we then took non-drinkers as the reference group and used the random-effect model to recalculate the pooled relative risks and 95% CIs of different alcohol consumption levels. Cochran's Q test and the I^2^ statistics are the tools to evaluate the heterogeneity between studies. I^2^ values equal to or exceeding 50% are usually judged to represent large heterogeneity. The Egger's test in which a *p* < 0.1 is taken as statistical evidence of the presence of small-study effects was used to calculate the publication bias (20). For all tests (except for the heterogeneity and small-study effects), *p* < 0.05 was considered statistically significant. All calculations were conducted with Stata 16.0.

## Results

### Search Results

**Figure 1:**
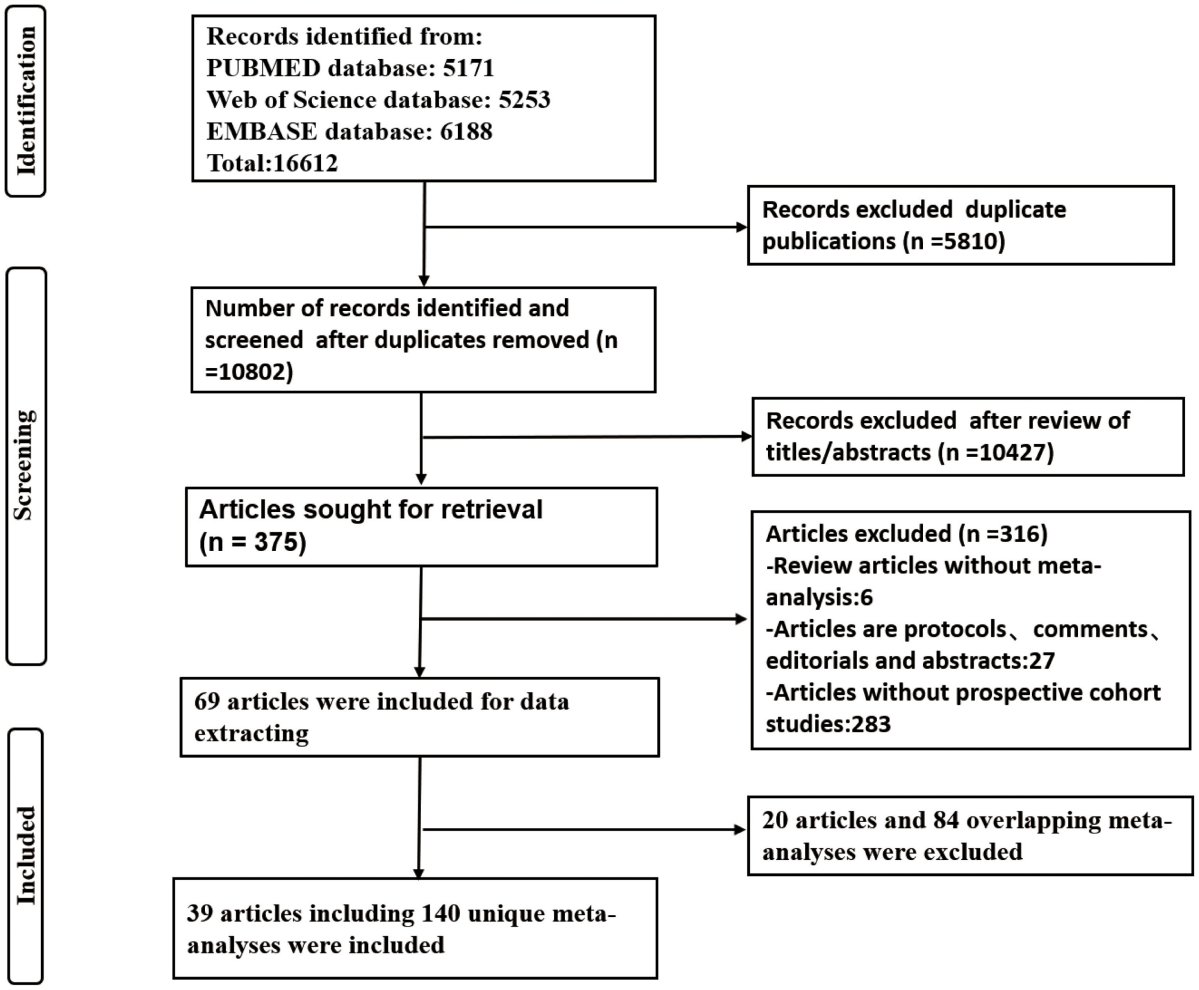
Flow diagram of literature search and study selection.

The results of systematic research and selection of eligible studies are shown in Figure 1. We included 59 publications with 224 meta-analyses (9, 21–78). Eighty-four meta-analyses showed overlapping results were removed (Supplementary Table 1), and finally 39 studies with 140 unique meta-analyses were retained, with 50, 47 and 43 unique meta-analysis results in the low, moderate and high alcohol consumption groups, respectively (Supplementary Table 2). The median number of studies included in meta-analyses was five (range 2–44), the median number of participants was 170,691 (range 842–3,702,738), and the median number of cases was 2,014 (range 85–104,278).

### Quality Assessment of Meta-Analyses

**Figure 2:**
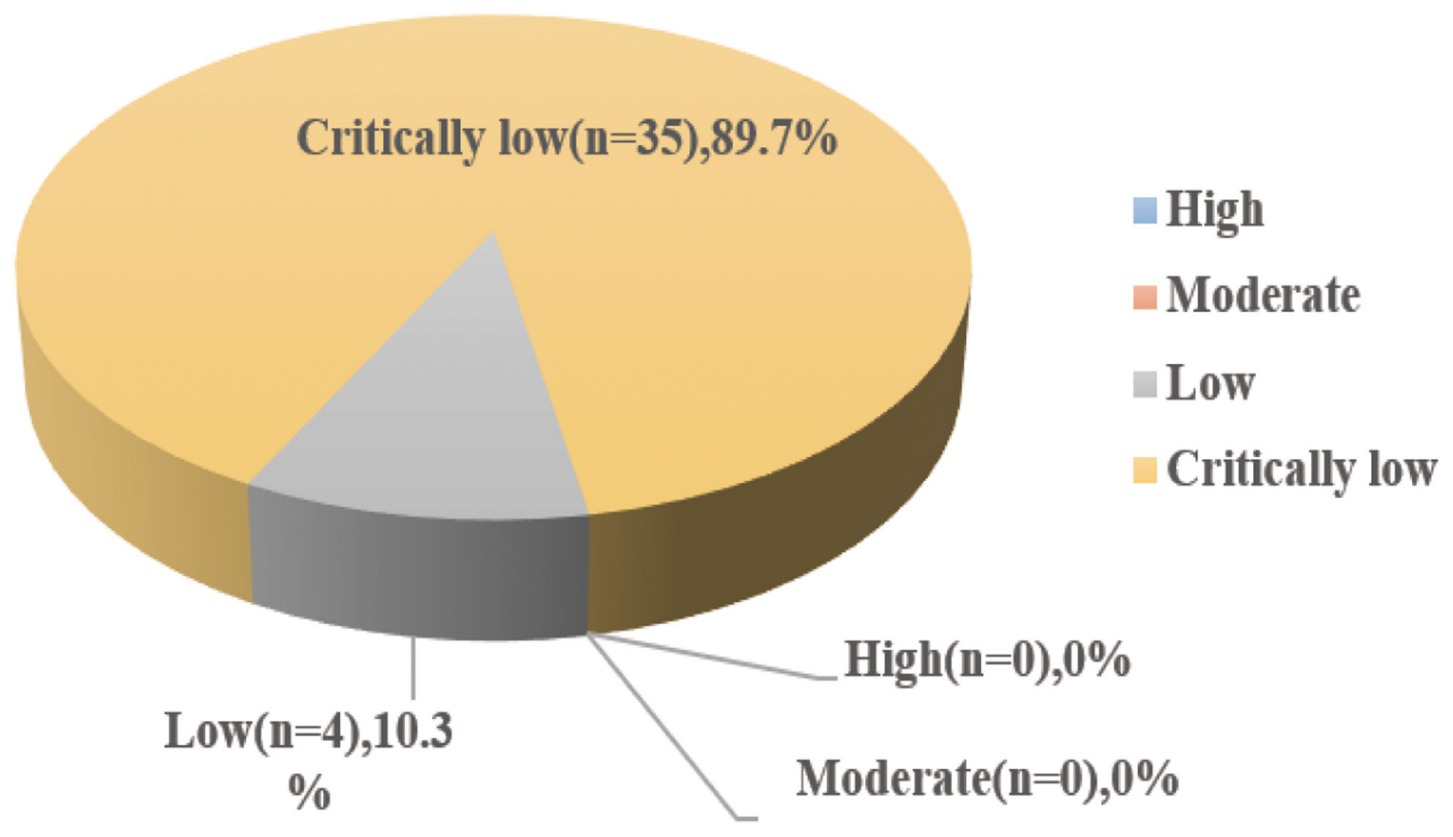
Map of results of AMSTAR 2.

The overall AMSTAR 2 scores of each included study are presented in Supplementary Table 3. Only 4 studies were rated as low methodological quality and the remaining 35 studies were all assessed to be critically low methodological. It is worthy to note that there has no high/moderate methodological quality based on the AMSTAR 2 strict criteria (Figure 2).

### Cancer Outcomes

**Figure 3:**
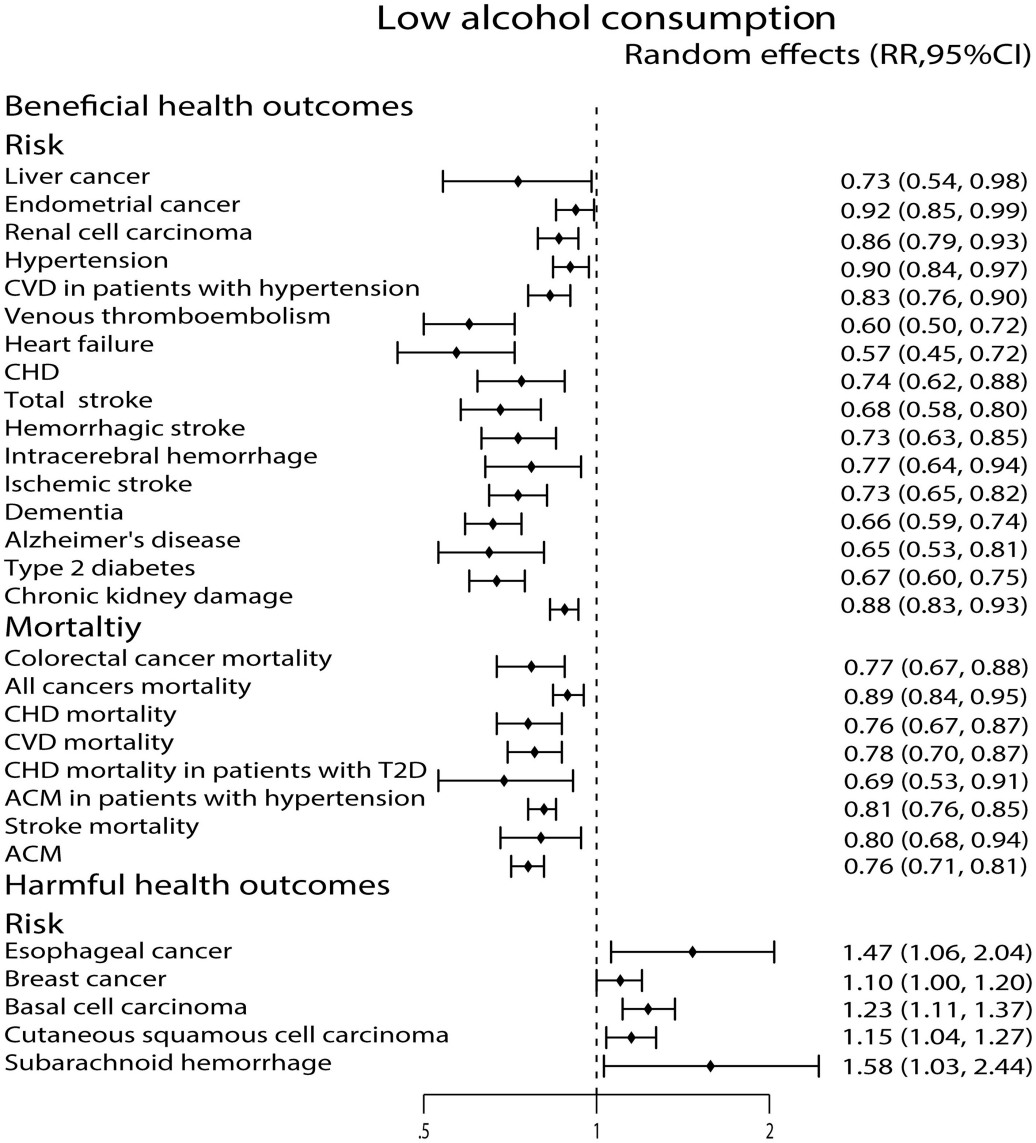
Forest plot: recalculated effects estimates of meta-analyses reporting significant associations of low alcohol consumption with health outcomes. RR, relative risk; CI, confidence interval; CVD, cardiovascular disease; CHD, coronary heart disease; ACM, all-cause mortality.

Compared with non-drinkers, low alcohol consumption decreased the risk of liver cancer (29), endometrial cancer (32) and renal cell carcinoma (72). However, low alcohol consumption increased the risk of esophageal cancer (73), breast cancer (22), cutaneous basal cell carcinoma (40) and cutaneous squamous cell carcinoma (40). Low alcohol consumption was also related to a 23% reduction in colorectal cancer mortality (43) and a 11% reduction in all cancers mortality (28) (Figure 3).

**Figure 4:**
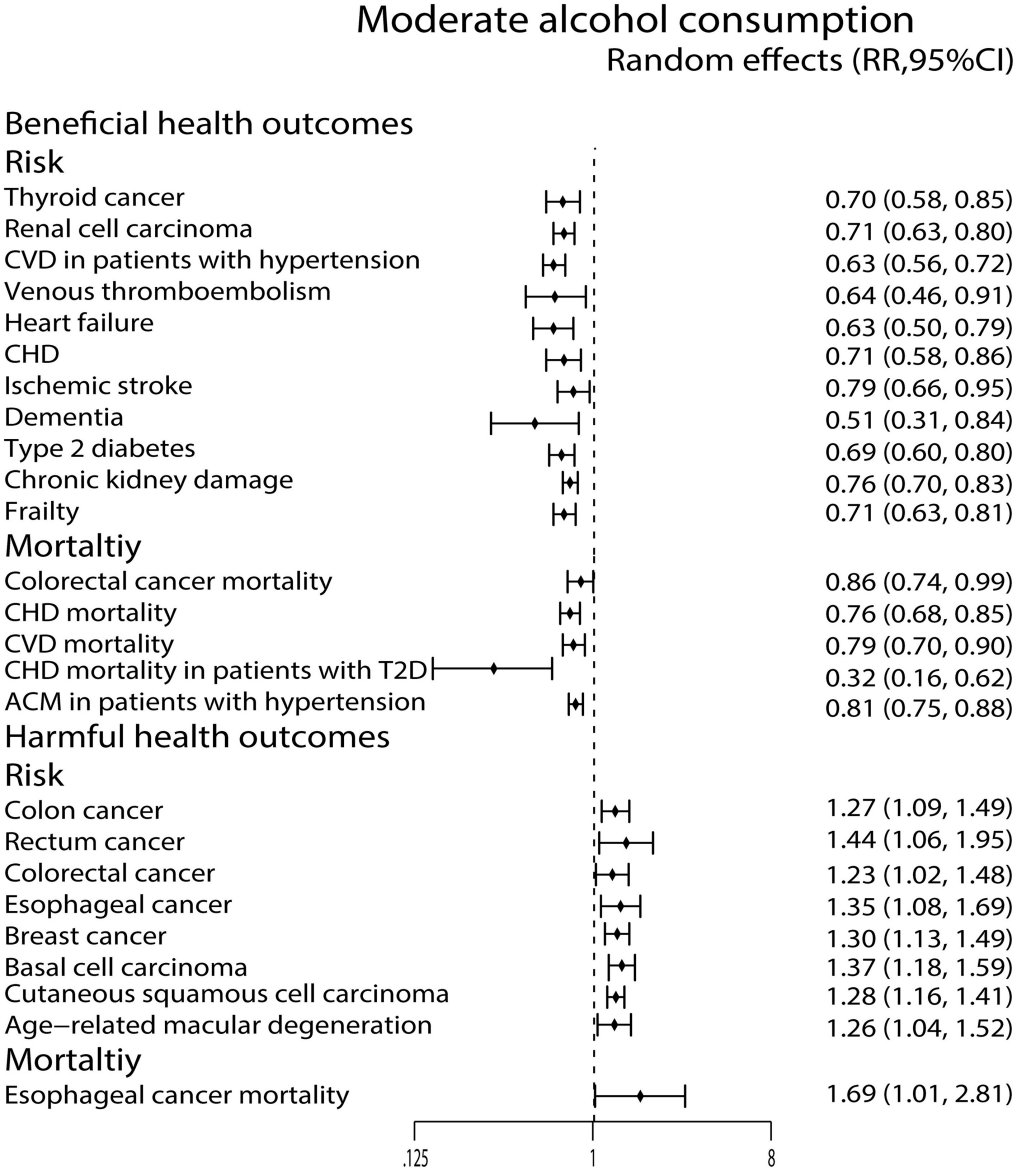
Forest plot: recalculated effects estimates of meta-analyses reporting significant associations of moderate alcohol consumption with health outcomes. RR, relative risk; CI, confidence interval; CHD, coronary heart disease; CVD, cardiovascular disease; ACM, all-cause mortality.

Moderate alcohol consumption was associated with a reduced risk of thyroid cancer (44) and renal cell carcinoma (72), while it showed increased risk of colon cancer (54), rectum cancer (54), colorectal cancer (54), esophageal cancer (73), breast cancer (22), cutaneous basal cell carcinoma (40) and cutaneous squamous cell carcinoma (40). Moreover, it reduced colorectal cancer mortality (43) and increased esophageal cancer mortality (53) (Figure 4).

**Figure 5:**
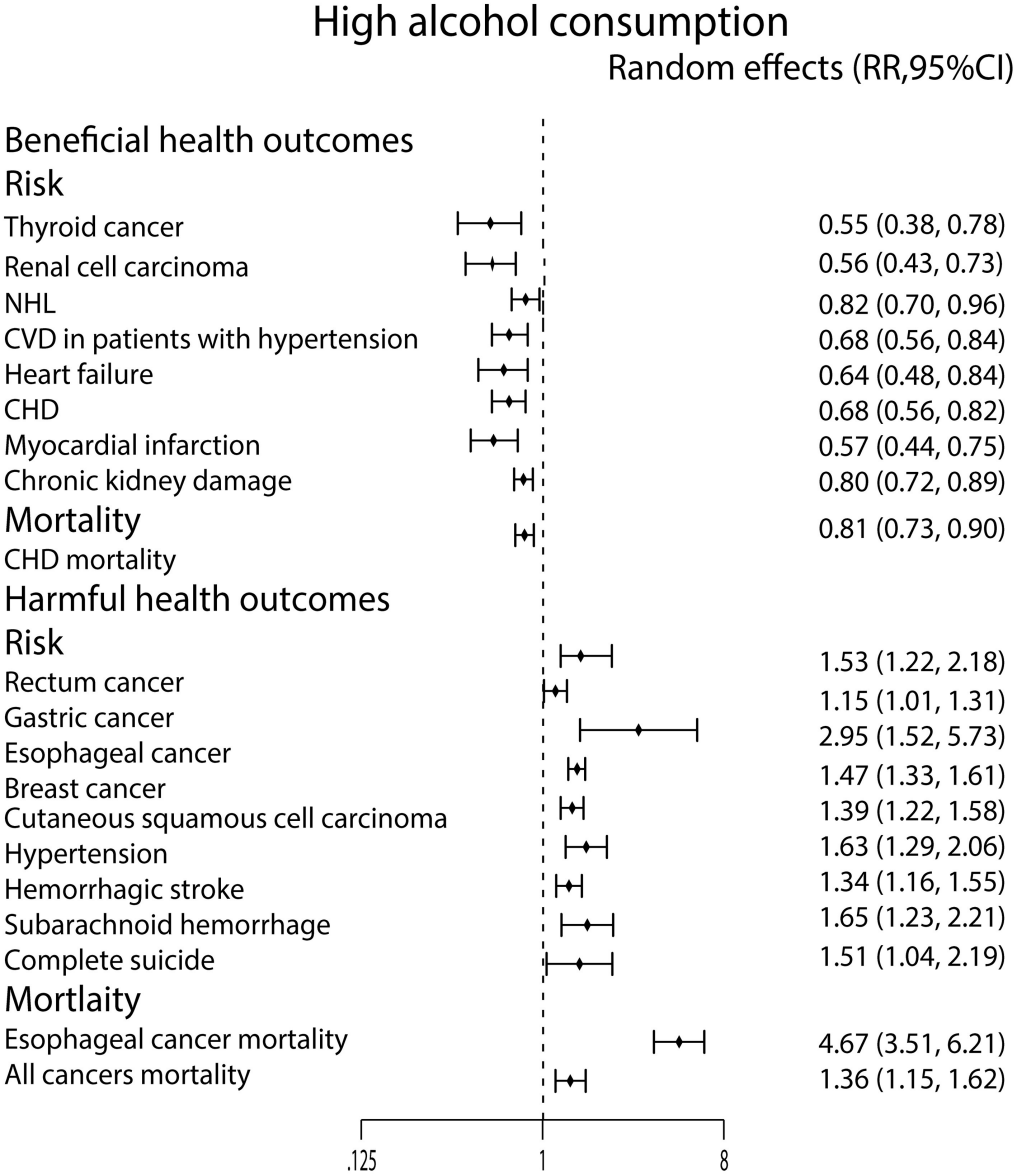
Forest plot: recalculated effects estimates of meta-analyses reporting significant associations of high alcohol consumption with health outcomes. RR, relative risk; CI, confidence interval; CVD, cardiovascular disease; CHD, coronary heart disease.

For high alcohol consumption, we found that it only decreased the incidence of thyroid cancer (44) and renal cell carcinoma (72), but it can increase the incidence of rectum cancer (54), gastric cancer (71), esophageal cancer (73), breast cancer (22), and cutaneous squamous cell carcinoma (40). In the meanwhile, high alcohol consumption was significantly related to esophageal cancer mortality (53) and all cancers mortality (28) (Figure 5).

### Hematological Malignancy Outcomes

We found only high alcohol consumption lowered the risk of NHL (55) (Figure 5).

### Circulatory System Outcomes

Low alcohol consumption lowered the risk of hypertension (59), CVD in patients with hypertension (68), venous thromboembolism (VTE) (52), heart failure (45), and CHD (9). In addition, low alcohol consumption was also related to a 24% reduction in CHD mortality (64), a 22% reduction in CVD mortality (9), a 31% reduction in CHD mortality in patients with T2D (49) and a 19% reduction in all-cause mortality (ACM) in patients with hypertension (68) (Figure 3).

We observed that the moderate alcohol consumption was related to a decreased risk of CVD in patients with hypertension (68), VTE (52), heart failure (45) and CHD (9). It also reduced the CHD mortality (64), CVD mortality (9), CHD mortality in patients with T2D (49) and ACM in patients with hypertension (68) (Figure 4).

Similarly, high alcohol consumption lowered the incidence of CVD in patients with hypertension (68), heart failure (45), CHD (9) and MI (70). What's more, it also lowered the CHD mortality (64). On the contrary, high alcohol consumption strongly increased the incidence of hypertension (59) (Figure 5).

### Nervous System Outcomes

As we see in Figure 3, low alcohol consumption strongly diminished the risk of total stroke (35), hemorrhagic stroke (35), intracerebral hemorrhage (ICH) (35), ischemic stroke (35), dementia (26) and Alzheimer's disease (AD) (26). We also found that low alcohol consumption was associated with a 20% reduction in stroke mortality (9). On the contrary, low alcohol consumption increased the risk of subarachnoid hemorrhage (SAH) (35) (Figure 3).

Moderate alcohol consumption only lowered ischemic stroke risk (35) and dementia risk (26) in this study (Figure 4). In contrast, the high alcohol consumption was associated with an increased risk of hemorrhagic stroke (35) and SAH (35) (Figure 5).

### All-Cause Mortality

Compared with non-drinkers, low alcohol consumption was associated with decreased ACM (63) (Figure 3).

### Metabolic Outcomes

Both low and moderate alcohol consumption had protective effects against developing type 2 diabetes (T2D) (25) (Figures 3, 4).

### Ophthalmic Outcomes

Moderate alcohol consumption was found to increase the AMD risk (27) (Figure 4).

### Other Health Outcomes

High alcohol consumption was associated with an increased risk of completes suicide (58) (Figure 5). For chronic kidney damage (23), the low, moderate and high alcohol consumption were all correlated to reduce the incidence (Figures 3–5). What's more, moderate alcohol consumption was found to strongly decrease the risk of frailty (21) in this study (Figure 4).

However, the low, moderate, and high drinking groups were not associated with 21, 22, and 23 health outcomes, respectively (Supplementary Figures 1–3).

### Strength of Epidemiologic Evidence

**Figure 6:**
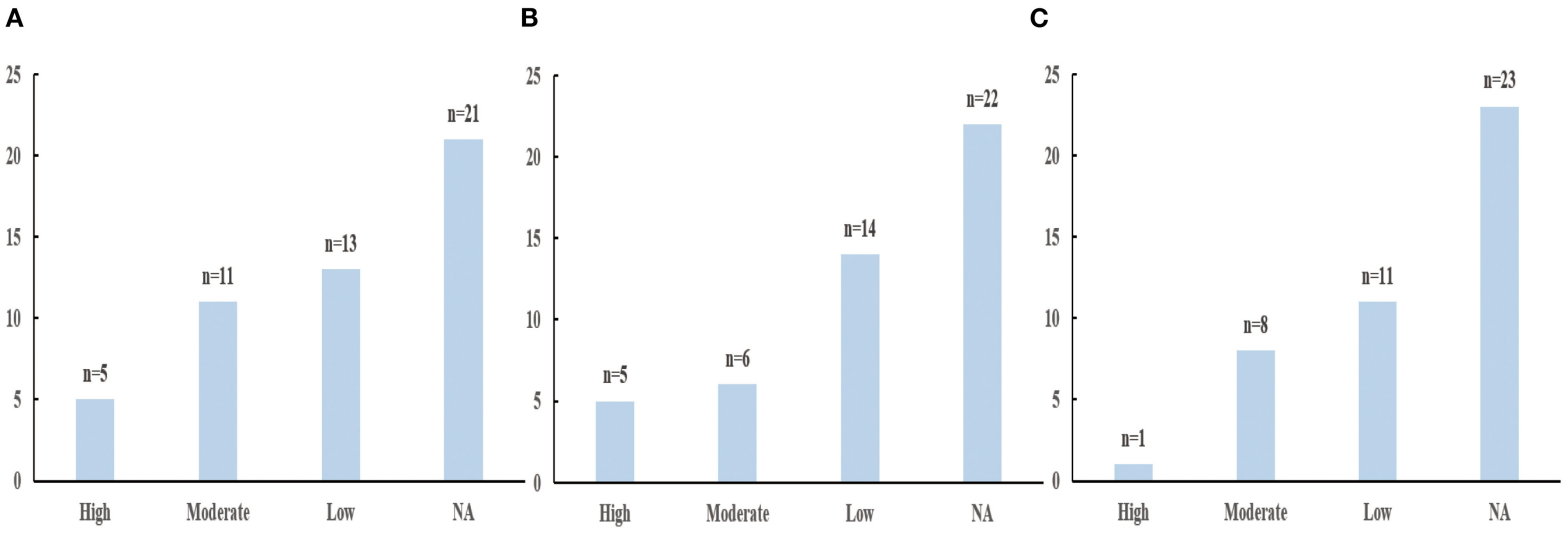
The results of evidecnce assessment. **(A)** Low alcohol consumption. **(B)** Moderate alcohol consumption. **(C)** High alcohol consumption.

Based on the criteria mentioned above, the assessment of epidemiologic evidence was not applicable for 21, 22, 23 health outcomes, respectively in low, moderate and high alcohol consumption because they show no statistically significant (*P* > 0.05) (Supplementary Table 4). Figure 6 showed the results of epidemiologic evidence of each group.

In low alcohol consumption group, five health outcomes (the decreased risk of renal cell carcinoma and dementia; the decreased mortality of colorectal cancer and all-cause in patients with hypertension; the increased risk of cutaneous basal cell carcinoma) showed high epidemiologic evidence; 11 health outcomes (the decreased risk of CVD in patients with hypertension, heart failure, total stroke, HS, ischemic stroke, T2D, and AD; the decreased mortality of all cancer, CHD, all-cause; the increased risk of breast cancer) showed moderate epidemiologic evidence; and 13 health outcomes showed weak epidemiologic evidence (Supplementary Table 5).

In moderate alcohol consumption group, five health outcomes (the decreased risk of renal cell carcinoma, CVD in patients with hypertension; the decreased mortality of all-cause in patients with hypertension; the increased risk of cutaneous basal cell carcinoma and cutaneous squamous cell carcinoma) showed high epidemiologic evidence; six health outcomes (the decreased risk of heart failure, CHD, T2D and frailty; the decreased mortality of CHD; the increased risk of breast cancer) showed moderate epidemiologic evidence; 14 health outcomes showed weak epidemiologic evidence (Supplementary Table 6).

In high alcohol consumption group, only one health outcome (the increased risk of hemorrhagic stroke) showed high epidemiologic evidence; eight health outcomes (the decreased risk of renal cell carcinoma, CVD in patients with hypertension, heart failure, CHD, and CKD; the decreased mortality of CHD; the increased risk of breast cancer and cutaneous squamous cell carcinoma) showed moderate epidemiologic evidence; 11 health outcomes showed weak epidemiologic evidence (Supplementary Table 7).

## Discussion

### Main Findings

We included 39 publications, which comprised 140 unique meta-analyses of prospective studies. We found the quality of evidence was graded as high for the four beneficial health outcomes (the decreased risk of renal cell carcinoma and dementia as well as the decreased mortality of colorectal cancer and all-cause in patients with hypertension) and one harmful health outcome (the increased risk of cutaneous basal cell carcinoma) in low alcohol consumption. For moderate alcohol consumption, the quality of evidence was graded as high for three beneficial health outcomes (the decreased risk of renal cell carcinoma, CVD in patients with hypertension; the decreased mortality of all-cause in patients with hypertension) and two harmful health outcomes (the increased risk of cutaneous basal cell carcinoma and cutaneous squamous cell carcinoma). In the high alcohol consumption, the quality of evidence was graded as high for only one harmful health outcome (the increased risk of hemorrhagic stroke) (Supplementary Tables 5–7).

### Comparison With Other Studies and Possible Explanations

According to ESMO Clinical Practice Guideline (79), low and moderate alcohol consumption (about 0.1–49.9 g alcohol per day) appears to have a protective effect for renal cell carcinoma. This information accords with our results that low and moderate alcohol consumption reduced the incidence of renal cell carcinoma with high epidemiologic evidence. The potential biologic mechanisms for this anti-cancer effect involve antioxidant phenolic compounds in alcohol, which can reduce oxidative stress and contribute to apoptosis by arresting the cell cycle (80, 81). Additionally, alcohol can reduce the time that carcinogenic solutes contact renal epithelial cells and control hypertension, which plays a role in protecting against renal cell carcinoma (82, 83). Furthermore, increased risk of renal cell carcinoma has been observed in individuals with diabetes or obesity and light to moderate alcohol consumption improved insulin sensitivity and lowered the risk of T2D thus lowering the incidence of renal cell carcinoma (16, 84, 85). Our umbrella review demonstrated that low alcohol consumption (includes wine, beer and liquor) reduced colorectal cancer mortality with high epidemiologic evidence. Related mechanisms may be involved in resveratrol and anthocyanin. Resveratrol found in grape skins can inhibit the occurrence, promotion and progression of tumors, and it has been found to have anti-proliferation effects *in vivo* and *in vitro* (86–89). Anthocyanin, which is presented in red wine (also in lower concentrations in white wine and beer), has been reported to have a suppressing effect on colon cancer cells *in vitro*, and phenolic acid and anthocyanin have been shown to inhibit colon cancer cell viability and to increase apoptosis (90–92).

However, these results should be interpreted carefully in a broader context because there was some robust evidence that alcohol increased the risk of oropharyngeal and larynx cancer, esophageal cancer, hepatocellular carcinoma, colon cancer and breast cancer by a statement of the American Society of Clinical Oncology in 2018 (6). It's worth noting that low/moderate alcohol consumption was related to the evaluated risk of cutaneous basal cell carcinoma and cutaneous squamous cell carcinoma with high epidemiologic evidence in our umbrella review, which was in accord with the above recommendation. Acetaldehyde, the terminal alcohol metabolite with carcinogenic and mutagenic effects by binding to DNA and protein, plays a key role in the pathophysiology of increasing cancer incidence (93–95). Ethanol-induced cancer also involves the induction of oncogenes or the suppression of tumor suppressor genes, which is also the main mechanism leading to cancers (95). Furthermore, the photosensitivity of alcohol metabolites can enhance cell damage and the immunosuppressive effect of alcohol (96, 97). Overall, although our results demonstrate a benefit of alcohol consumption for the risk of renal cell carcinoma and colorectal cancer mortality, caution should be taken in translating these results into guideline recommendations.

The Dietary Guidelines Advisory Committee (US) stated in 2020 (98), if alcohol is consumed, it should be consumed in moderation (≤ 1 and 2 drinks (about 12.5~25 g alcohol) /day for women and men, respectively) and only by adults of legal drinking age. A large prospective cohort of >330,000 adults emphasizes that light to moderate alcohol consumption (0.1~196 g/week for men; 0.1~98 g/week for women) reduced the all-cause mortality by up to 29% and CVD mortality by ≤ 24% in the US (99). Similarly, in our study, low/moderate alcohol consumption reduced CVD events and all-cause mortality in hypertensive patients, with high epidemiologic evidence. This is consistent with the above studies and dietary guidelines. The causal relationship between regular low alcohol consumption and prevention of CVD was supported by some biomedical evidence, which is mediated by anti-inflammatory effects, reduction of fibrinogen levels, inhibition of platelet activation and increased high-density lipoprotein level by terminal alcohol metabolite (100–102). The beneficial effects of alcohol on CVD account for 77.8% of the total beneficial effects of alcohol (103), and ACM includes deaths caused by CVD, so it is reasonable that low/moderate alcohol consumption leads to a decrease in ACM in hypertensive patients.

Our study indicated that high alcohol consumption increased the risk of hemorrhagic stroke with high epidemiologic evidence. Likewise, the *ad-hoc* Working Group Of The Italian Society Of Human Nutrition (104) suggests that alcohol intake should be limited to 1 drink per day for women and to 2 drinks per day for men to avoid ischemic and hemorrhagic stroke caused by heavy drinking. Alcohol has a detrimental effect on platelet function and platelet count, affecting platelet aggregation and thus damaging human hemostasis (105, 106). Overusing of alcohol may increase the hemorrhage risk linked to small-vessel vasculopathy (107). In addition, the adverse impact of alcohol consumption on blood pressure may directly increase the risk of hemorrhagic stroke. Therefore, controlling alcohol consumption is important for preventing hemorrhagic stroke.

Recently, an umbrella review (108), including 14 observational studies and RCTs, indicated that alcohol intake was a protective factor for dementia with weak evidence. Similarly, our prospective umbrella review, showing low alcohol consumption decreased the incidence of dementia with high epidemiologic evidence, seems to be more robust. Alcohol increases cerebral blood flow, reduces clotting, increases antithrombotic activity, and increases endothelium dilation, which has protective effects against atherosclerosis, vascular occlusion, and cerebral hypoperfusion (109, 110). But, WHO guideline in 2019 (111) suggests that interventions aimed at reducing or ceasing hazardous and harmful drinking should be offered to adults with normal cognition and mild cognitive impairment to reduce the risk of dementia because there is extensive evidence on excessive alcohol as a risk factor for dementia (112, 113). Therefore, while low alcohol intake is a protective factor for dementia, considering the harmful effects of heavy alcohol intake on dementia and other diseases, we are more cautious about incorporating these results into recommendations.

### Strength and Limitation

Our umbrella review had the following strengths. On the one hand, to our knowledge, it was the first umbrella review of prospective meta-analyses about the associations between alcohol consumption and health outcomes at present. The included primary studies were all based on prospective observational study design so that the recall bias can be reduced to a certain extent. On the other hand, we used validated tools to evaluate the methodological quality and quality of epidemiological evidence in our umbrella review. All meta-analyses included in this review had high or moderate methodological quality. What's more, we unified the alcohol grouping criteria and recalculated effect sizes, heterogeneity, and small-study effects better to explain the association between alcohol and health outcomes.

However, there were several limitations in our umbrella review. Firstly, we only included the prospective meta-analyses and therefore, we may have missed other health outcomes not yet studied through prospective meta-analysis. Secondly, we did not explore the different types of alcoholic beverages in this study. Arranz et al. (114) pointed out that significant inverse association between regular and moderate wine consumption and vascular risk, particularly red wine, and a similar relationship is reported for beer consumption, while lower protection is described for the consumption of any spirituous beverage. A cross-sectional study showed that compared to never drinkers of each type of alcoholic drink, red wine, champagne/white wine and fortified wine drinkers had a lower BMI, whereas beer and spirits drinkers had higher BMI compared to never drinkers of each type of alcoholic drink (115). Another study found that liquor consumption and binge drinking was associated with increased risk of VTE, whereas wine consumption was possibly associated with reduced risk of VTE (116). However, due to the lack of raw data, there is little literature to explore the association between various health outcomes and different types of alcoholic beverages. In addition, most of the primary studies have not reported the alcoholic type and we failed to conduct a subgroup based on the alcoholic different alcoholic drink types. Thirdly, we did not evaluated quality of the primary studies, since it was beyond the scope of the current umbrella review. Finally, we did not explore the subgroup analysis or sensitivity analysis (e.g., by sex, geography, or other factors that can influence the result).

## Conclusion

In summary, the data presented in this study demonstrated that there were 49 beneficial associations and 25 harmful associations with nominally statistically significant summary results in 140 health outcomes for low/moderate alcohol consumption, while harmful associations mainly related to hemorrhagic stroke, hypertension, and cancers for high alcohol consumption. However, the quality of evidence was rated high only for seven beneficial associations (renal cell carcinoma risk, dementia risk, colorectal cancer mortality, and ACM in patients with hypertension for low alcohol consumption; renal cell carcinoma risk, CVD risk in patients with hypertension, and ACM in patients with hypertension for moderate consumption) and four harmful associations (cutaneous basal cell carcinoma risk for low alcohol consumption; cutaneous basal cell carcinoma risk and cutaneous squamous cell carcinoma risk for moderate alcohol consumption; hemorrhagic stroke risk for high alcohol consumption). This reminds us that we should drink in moderation and avoid binge drinking or heavy drinking. To achieve high quality of evidence for the associations of alcohol with the health outcomes and give strong recommendations, more robust and larger prospective studies are needed.

## Data Availability Statement

The original contributions presented in the study are included in the article/Supplementary Material, further inquiries can be directed to the corresponding author.

## Author Contributions

LZ, WC, TW, and ST contributed to the conception and design of the umbrella review. LZ, WC, TW, QZ, LL, JLa, and JLi were involved in the acquisition and analysis of the data. LZ and TW interpreted the results. LZ and ST drafted the manuscript. All authors read and approved the final manuscript.

## Funding

This study was supported by the Special project for high-level talents of the Affiliated Hospital of Youjiang Medical University for Nationalities, in Baise, China (No. R202011703).

## Conflict of Interest

The authors declare that the research was conducted in the absence of any commercial or financial relationships that could be construed as a potential conflict of interest.

## Publisher's Note

All claims expressed in this article are solely those of the authors and do not necessarily represent those of their affiliated organizations, or those of the publisher, the editors and the reviewers. Any product that may be evaluated in this article, or claim that may be made by its manufacturer, is not guaranteed or endorsed by the publisher.

## Abbreviations

CVD: Cardiovascular disease
ACM: all-cause mortality
CHD: coronary heart disease
MI: myocardial infarction
SAH: subarachnoid hemorrhage
ICH: intracerebral hemorrhage
VTE: venous thromboembolism
NHL: non-Hodgkin's lymphoma
T2D: type 2 diabetes mellitus
AD: Alzheimer's disease
RR: relative risk
OR: odd ratio
HR: hazard ratio
CI: confidence interval.
