# Supplementary material for: Alcohol and Health Outcomes: An Umbrella Review of Meta-Analyses Base on Prospective Cohort Studies
Source: Front Public Health. 2022 May 4;10:859947. doi: 10.3389/fpubh.2022.859947 (PMC9115901; doi:10.3389/fpubh.2022.859947)
Supplement: Supplementary file 1 [file Table_1.docx]

**ESM Table 1.** General characteristics and main findings of the excluded **84 meta-analyses**

| Health outcome | Author, year | Studies | Subjects | Cases | Comparison (vs non-drinkers) | Effect size | | Heterogeneity | | Small-study effect |
| --- | --- | --- | --- | --- | --- | --- | --- | --- | --- | --- |
|  |  |  |  |  |  | Relative risk and 95% CIs | P-value | I^2^ | P-value |  |
| **Cancers** |  |  |  |  |  |  |  |  |  |  |
| **Risk** |  |  |  |  |  |  |  |  |  |  |
| Esophageal cancer^1^ | Islami et al, 2011 | 4 | 946882 | 711 | Low | 0.03(0.55-1.14) | 0.214 | 79.70% | 0.001 | 0.428 |
| Esophageal cancer^1^ | Islami et al, 2011 | 5 | 757344 | 547 | Moderate | 1.17(0.65-2.13) | 0.602 | 90.10% | 0.000 | 0.111 |
| Esophageal cancer^1^ | Islami et al, 2011 | 4 | 532577 | 485 | High | 2.40(1.47-3.93) | 0.000 | 85.60% | 0.000 | 0.073 |
| Endometrial cancer^2^ | Friberg et al, 2010 | 4 | 925471 | 4368 | Low | 0.90(0.82-0.98) | 0.021 | 45.50% | 0.102 | 0.120 |
| Endometrial cancer^2^ | Friberg et al, 2010 | 3 | 489959 | 2269 | Moderate | 0.88(0.62-1.35) | 0.550 | 77.10% | 0.013 | 0.135 |
| Endometrial cancer^2^ | Friberg et al, 2010 | 2 | 138919 | 1585 | High | 1.19(0.45-3.17) | 0.724 | 94.50% | 0.000 | NA |
| Endometrial cancer^3^ | Sun et al, 2011 | 4 | 154519 | 1300 | Low | 1.00(0.88-1.13) | 0.978 | 0.00% | 0.925 | 0.398 |
| Endometrial cancer^3^ | Sun et al, 2011 | 3 | 50281 | 502 | Moderate | 1.11(0.79-1.56) | 0.851 | 0.00% | 0.551 | NA |
| **Circulatory system disease** |  |  |  |  |  |  |  |  |  |  |
| **Risk** |  |  |  |  |  |  |  |  |  |  |
| Atrial fibrillation^4^ | Larsson et al, 2014 | 4 | 66754 | 2023 | Low | 0.79(0.45-1.37) | 0.395 | 96.90% | 0.000 | 0.441 |
| Atrial fibrillation^4^ | Larsson et al, 2014 | 4 | 63207 | 2673 | Moderate | 1.06(0.94-1.19) | 0.333 | 24.20% | 0.260 | 0.598 |

**(*continued*)**

| Health outcome | Author, year | Studies | Subjects | Cases | Comparison (vs non-drinkers) | Effect size | | Heterogeneity | | Small-study effect |
| --- | --- | --- | --- | --- | --- | --- | --- | --- | --- | --- |
|  |  |  |  |  |  | Relative risk and 95% CIs | P-value | I^2^ | P-value |  |
| Atrial fibrillation^4^ | Larsson et al, 2014 | 6 | 78832 | 4315 | High | 1.20(085-1.70) | 0.294 | 93.60% | 0.000 | 0.096 |
| Hypertension^5^ | Jung et al, 2020 | 3 | 4915 | 1032 | Low | 1.14(0.95-1.36) | 0.148 | 0.00% | 0.923 | NA |
| Hypertension^5^ | Jung et al, 2020 | 4 | 4311 | 860 | Moderate | 1.13(0.83-1.52) | 0.434 | 72.20% | 0.027 | 0.311 |
| Hypertension^5^ | Jung et al, 2020 | 5 | 5485 | 1611 | High | 2.31(1.93-2.78) | 0.000 | 29.80% | 0234 | 0.770 |
| Heart failure^6^ | Padilla et al, 2010 | 3 | 115517 | 3151 | Low | 0.74(0.57-0.95) | 0.019 | 94.00% | 0.000 | 0.242 |
| Heart failure^6^ | Padilla et al, 2010 | 4 | 48706 | 1781 | Moderate | 0.66(0.57-0.76) | 0.000 | 24.60% | 0.246 | 0.268 |
| Heart failure^6^ | Padilla et al, 2010 | 3 | 44409 | 1703 | High | 0.72(0.62-0.83) | 0.000 | 12.40% | 0.331 | 0.801 |
| Heart failure^7^ | Larsson et al, 2016 | 3 | 48706 | 1781 | Low | 0.74(0.57-0.95) | 0.019 | 94.00% | 0.000 | 0.242 |
| Heart failure^7^ | Larsson et al, 2016 | 4 | 45098 | 2617 | Moderate | 0.66(0.57-0.76) | 0.000 | 24.60% | 0.264 | 0.268 |
| Heart failure^7^ | Larsson et al, 2016 | 3 | 44409 | 1703 | High | 0.72(0.62-0.83) | 0.000 | 12.40% | 0.331 | 0.801 |
| CHD^8^ | Liu et al, 2010 | 3 | 51107 | 900 | Low | 0.72(0.38-1.36) | 0.032 | 70.80% | 0.114 | 0.613 |
| CHD^8^ | Liu et al, 2010 | 4 | 59931 | 970 | Moderate | 0.46(0.20-1.05) | 0.066 | 87.80% | 0.00` | 0.648 |
| CHD^8^ | Liu et al, 2010 | 4 | 53575 | 858 | High | 0.50(0.27-0.92) | 0.026 | 72.70% | 0.026 | 0.801 |
| CHD^9^ | Yang et al,2016 | 6 | 82931 | 3003 | Low | 0.90(0.82-0.98) | 0.020 | 0.00% | 0.499 | 0.423 |

**(*continued*)**

| Health outcome | Author, year | Studies | Subjects | Cases | Comparison (vs non-drinkers) | Effect size | | Heterogeneity | | Small-study effect |
| --- | --- | --- | --- | --- | --- | --- | --- | --- | --- | --- |
|  |  |  |  |  |  | Relative risk and 95% CIs | P-value | I^2^ | P-value |  |
| CHD^9^ | Yang et al,2016 | 4 | 19171 | 1224 | Moderate | 0.55(0.33-0.92) | 0.022 | 85.00% | 0.000 | 0.644 |
| CHD^9^ | Yang et al,2016 | 6 | 79518 | 1557 | High | 0.49(0.39-0.61) | 0.000 | 36.30% | 0.151 | 0.334 |
| CHD^10^ | Yoonet al, 2020 | 2 | 10317 | 268 | Low | 1.03(0.67-1.58) | 0.889 | 65.90% | 0.053 | NA |
| CHD^10^ | Yoonet al, 2020 | 2 | 9431 | 246 | Moderate | 1.02(0.75-1.38) | 0.918 | 0.00% | 0.491 | NA |
| CHD^10^ | Yoonet al, 2020 | 3 | 11050 | 386 | High | 1.35(1.02-1.79) | 0.036 | 33.70% | 0.210 | 0.731 |
| **Mortality** |  |  |  |  |  |  |  |  |  |  |
| CHD mortality^11^ | Ronksley et al, 2011 | 14 | 492008 | 22120 | Low | 0.71(0.65-0.78) | 0.000 | 85.60% | 0.000 | 0.321 |
| CHD mortality^11^ | Ronksley et al, 2011 | 19 | 370922 | 18282 | Moderate | 0.78(0.69-0.88) | 0.000 | 86.40% | 0.000 | 0.523 |
| CHD mortality^11^ | Ronksley et al, 2011 | 14 | 317165 | 17280 | High | 0.79(0.69-0.92) | 0.002 | 92.20% | 0.000 | 0.144 |
| CHD mortality^8^ | Liu et al, 2010 | 2 | 59627 | 655 | Low | 0.96(0.61-1.50) | 0.848 | 75.70% | 0.042 | NA |
| CHD mortality^8^ | Liu et al, 2010 | 4 | 77252 | 852 | Moderate | 0.74(0.57-0.96) | 0.011 | 45.30% | 0.139 | 0.138 |
| CHD mortality^8^ | Liu et al, 2010 | 4 | 99129 | 900 | High | 0.93(0.42-2.03) | 0.854 | 81.50% | 0.001 | 0.429 |
| **Nervous system outcomes** |  |  |  |  |  |  |  |  |  |  |
| **Risk** |  |  |  |  |  |  |  |  |  |  |

**(*continued*)**

| Health outcome | Author, year | Studies | subjects | Cases | Comparison (vs non-drinkers) | Effect size | | Heterogeneity | | Small-study effect |
| --- | --- | --- | --- | --- | --- | --- | --- | --- | --- | --- |
|  |  |  |  |  |  | Relative risk and 95% CIs | P-value | I^2^ | P-value |  |
| Total stroke^11^ | Ronksley et al, 2011 | 5 | 65416 | 3058 | Low | 0.86(0.76-0.97) | 0.015 | 24.90% | 0.239 | 0.802 |
| Total stroke^11^ | Ronksley et al, 2011 | 4 | 58787 | 2954 | Moderate | 0.98(0.90-1.07) | 0.643 | 0.00% | 0.546 | 0.837 |
| Total stroke^11^ | Ronksley et al, 2011 | 7 | 123662 | 4039 | High | 1.13(1.02-1.24) | 0.014 | 30.00% | 0.169 | 0.901 |
| Total stroke^12^ | Zhang et al, 2014 | 6 | 197919 | 3861 | Low | 0.80(0.68-0.94) | 0.006 | 72.30% | 0.000 | 0.253 |
| Total stroke^12^ | Zhang et al, 2014 | 6 | 113951 | 4111 | Moderate | 1.01(0.92-1.11) | 0.814 | 24.30% | 0.244 | 0.798 |
| Total stroke^12^ | Zhang et al, 2014 | 7 | 119311 | 4012 | High | 1.17(1.06-1.29) | 0.002 | 33.50% | 0.141 | 0.898 |
| Hemorrhagic stroke^12^ | Zhang et al, 2014 | 5 | 206510 | 1101 | Low | 0.78(0.68-0.90) | 0.000 | 0.00% | 0.609 | 0.041 |
| Hemorrhagic stroke^12^ | Zhang et al, 2014 | 4 | 109851 | 997 | Moderate | 1.09(0.95-1.26) | 0.270 | 0.00% | 0.431 | 0.142 |
| Hemorrhagic stroke^12^ | Zhang et al, 2014 | 3 | 102984 | 950 | High | 1.29(1.02-1.62) | 0.032 | 36.10% | 0.166 | 0.522 |
| Intracerebral hemorrhage^12^ | Zhang et al, 2014 | 2 | 96449 | 256 | Low | 0.80(0.63-1.02) | 0.075 | 3.40% | 0.355 | NA |
| Intracerebral hemorrhage^12^ | Zhang et al, 2014 | 2 | 46153 | 182 | Moderate | 0.92(0.67-1.26) | 0.597 | 0.00% | 0.371 | NA |
| Intracerebral hemorrhage^12^ | Zhang et al, 2014 | 3 | 38437 | 756 | High | 1.22(0.93-1.62) | 0.156 | 32.00% | 0.196 | 0.664 |
| Ischemic stroke^12^ | Zhang et al, 2014 | 9 | 227246 | 2988 | Low | 0.80(0.71-0.93) | 0.002 | 48.20% | 0.019 | 0.235 |
| Ischemic stroke^12^ | Zhang et al, 2014 | 7 | 110678 | 2449 | Moderate | 0.91(0.75-1.10) | 0.314 | 60.90% | 0.018 | 0.210 |

**(*continued*)**

| Health outcome | Author, year | Studies | subjects | Cases | Comparison (vs non-drinkers) | Effect size | | | Heterogeneity | | Small-study effect | |
| --- | --- | --- | --- | --- | --- | --- | --- | --- | --- | --- | --- | --- |
|  |  |  |  |  |  | Relative risk and 95% CIs | | P-value | I^2^ | P-value |  |  |
| Ischemic stroke^12^ | Zhang et al, 2014 | 11 | 128200 | 2430 | High | | 1.10(0.95-1.26) | 0.216 | 46.40% | 0.045 | 0.442 | |
| Subarachnoid hemorrhage^12^ | Zhang et al, 2014 | 2 | 96449 | 100 | Low | | 0.93(0.63-1.39) | 0.737 | 0.00% | 0.441 | NA | |
| Subarachnoid hemorrhage^12^ | Zhang et al, 2014 | 2 | 46153 | 68 | Moderate | 1.29(0.76-2.17) | | 0.348 | 0.00% | 0.749 | NA | |
| Subarachnoid hemorrhage^12^ | Zhang et al, 2014 | 2 | 41082 | 100 | High | 1.86(1.13-2.53) | | 0.013 | 0.00% | 0.653 | NA | |
| Parkinson’s disease^13^ | Jiménez et,al 2019 | 4 | 412052 | 1239 | Low | 0.97(0.78-1.21) | | 0.783 | 55.40% | 0.081 | 0.341 |  |
| Parkinson’s disease^13^ | Jiménez et,al 2019 | 3 | 156806 | 565 | Moderate | 0.94(0.70-1.27) | | 0.695 | 43.80% | 0.169 | NA |  |
| Parkinson’s disease^13^ | Jiménez et,al 2019 | 3 | 151342 | 567 | High | 0.87(0.61-1.26) | | 0.467 | 46.20% | 0.156 | NA |  |
| **Mortality** |  |  |  |  |  |  | |  |  |  |  | |
| Stroke mortality^12^ | Zhang et al, 2014 | 6 | 236263 | 2202 | Low | 0.74(0.63-0.87) | | 0.000 | 12.50% | 0.335 | 0.770 | |
| Stroke mortality^12^ | Zhang et al, 2014 | 5 | 154115 | 1068 | Moderate | 0.93(0.70-1.24) | | 0.622 | 46.70% | 0.111 | 0.685 | |
| Stroke mortality^12^ | Zhang et al, 2014 | 5 | 198026 | 2248 | High | 1.27(0.99-1.63) | | 0.059 | 70.10% | 0.001 | 0.481 | |
| ACM^11^ | Ronksley et al, 2011 | 12 | 356231 | 39502 | Low | 0.79(0.72-0.88) | | 0.000 | 92.10% | 0.000 | 0.173 | |
| ACM^11^ | Ronksley et al, 2011 | 13 | 270762 | 37294 | Moderate | 0.84(0.75-0.95) | | 0.006 | 92.40% | 0.000 | 0.992 | |
| ACM^11^ | Ronksley et al, 2011 | 12 | 261636 | 39437 | High | 1.13(0.99-1.29) | | 0.066 | 97.30% | 0.000 | 0.867 | |

**(*continued*)**

| Health outcome | Author, year | Studies | subjects | Cases | Comparison (vs non-drinkers) | | Effect size | | | Heterogeneity | | | | | Small-study effect |
| --- | --- | --- | --- | --- | --- | --- | --- | --- | --- | --- | --- | --- | --- | --- | --- |
|  |  |  |  |  |  |  | Relative risk and 95% CIs | P-value | | I^2^ | | P-value | | |  |
| ACM^14^ | Jayasekara et al, 2014 | 3 | 18776 | 7855 | Low | 0.92(0.88-0.96) | | | 0.000 | | 0.00% | | 0.572 | 0.280 | |
| ACM^14^ | Jayasekara et al, 2014 | 3 | 12745 | 6428 | Moderate | 0.85(0.81-0.91) | | | 0.000 | | 0.00% | | 0.823 | 0.117 | |
| ACM^14^ | Jayasekara et al, 2014 | 4 | 12575 | 6231 | High | | 0.92(0.79-1.06) | 0.239 | | 77.80% | | 0.000 | | | 0.658 |
| ACM^15^ | Wang et al, 2014 | 9 | 44518 | 16212 | Low | | 0.89(0.82-0.96) | 0.002 | | 59.50% | | 0.006 | | | 0.674 |
| ACM^15^ | Wang et al, 2014 | 9 | 48901 | 9349 | Moderate | | 0.96(0.86-1.06) | 0.419 | | 57.10% | | 0.007 | | | 0.525 |
| ACM^15^ | Wang et al, 2014 | 9 | 46039 | 9415 | High | | 1.07(0.93-1.23) | 0.350 | | 81.70% | | 0.000 | | | 0.035 |
| ACM^16^ | Scd et al, 2006 | 15 | 328847 | 43117 | Low | | 0.82(0.75-0.90) | 0.000 | | 89.60% | | 0.000 | | | 0.022 |
| ACM^16^ | Scd et al, 2006 | 17 | 326140 | 42219 | Moderate | | 0.95(0.85-1.07) | 0.389 | | 93.30% | | 0.000 | | | 0.283 |
| ACM^16^ | Scd et al, 2006 | 20 | 327772 | 42751 | High | | 1.19(1.05-1.34) | 0.005 | | 95.90% | | 0.000 | | | 0.884 |
| **Metabolic outcomes** |  |  |  |  |  | |  |  | |  | |  | | |  |
| **Risk** |  |  |  |  |  | |  |  | |  | |  | | |  |
| Type 2 diabetes^17^ | Baliunas et al, 2009 | 16 | 256815 | 6820 | Low | | 0.75(0.68-0.84) | 0.000 | | 73.30% | | 0.000 | | | 0.896 |
| Type 2 diabetes^17^ | Baliunas et al, 2009 | 15 | 130587 | 4274 | Moderate | | 0.66(0.57-0.76) | 0.000 | | 73.70% | | 0.000 | | | 0.707 |
| Type 2 diabetes^17^ | Baliunas et al, 2009 | 13 | 126783 | 3503 | High | | 0.82(0.66-1.02) | 0.081 | | 87.20% | | 0.000 | | | 0.751 |

**(*continued*)**

| Health outcome | Author, year | Studies | subjects | Cases | Comparison (vs non-drinkers) | Effect size | | Heterogeneity | | Small-study effect |
| --- | --- | --- | --- | --- | --- | --- | --- | --- | --- | --- |
|  |  |  |  |  |  | Relative risk and 95% CIs | P-value | I^2^ | P-value |  |
| Type 2 diabetes^18^ | Han et al, 2020 | 5 | 105716 | 2276 | Low | 0.80(0.73-0.88) | 0.000 | 15.40% | 0.305 | 0.375 |
| Type 2 diabetes^18^ | Han et al, 2020 | 5 | 46297 | 1788 | Moderate | 0.72(0.57-0.92) | 0.009 | 67.80% | 0.014 | 0.284 |
| Type 2 diabetes^18^ | Han et al, 2020 | 7 | 33281 | 1879 | High | 1.19(1.08-1.31) | 0.000 | 0.00% | 0.503 | 0.449 |
| Type 2 diabetes^19^ | Koppes et al, 2005 | 9 | 163043 | 4683 | Low | 0.71(0.61-0.82) | 0.000 | 81.70% | 0.000 | 0.391 |
| Type 2 diabetes^19^ | Koppes et al, 2005 | 8 | 65945 | 2427 | Moderate | 0.64(0.56-0.73) | 0.000 | 53.40% | 0.036 | 0.927 |
| Type 2 diabetes^19^ | Koppes et al, 2005 | 9 | 58353 | 2474 | High | 0.85(0.69-1.03) | 0.097 | 80.70% | 0.000 | 0.851 |
| Type 2 diabetes^20^ | Huang et al, 2017 | 4 | 37538 | 1248 | Low | 0.74(0.59-0.92) | 0.008 | 44.10% | 0.128 | 0.430 |
| Type 2 diabetes^20^ | Huang et al, 2017 | 5 | 34578 | 1137 | Moderate | 0.73(0.57-0.94) | 0.016 | 51.10% | 0.041 | 0.095 |
| Type 2 diabetes^20^ | Huang et al, 2017 | 4 | 26546 | 758 | High | 1.04(0.60-1.81) | 0.892 | 86.10% | 0.000 | 0.869 |
| **Pregnancy and childhood outcome** |  |  |  |  |  |  |  |  |  |  |
| **Risk** |  |  |  |  |  |  |  |  |  |  |
| Low birth weight^21^ | Pereira et al, 2019 | 2 | 5654 | 312 | Low | 076(0.55-1.04) | 0.084 | 0.00% | 0.418 | NA |

CVD, cardiovascular disease; ACM, all-cause mortality; CHD, coronary heart disease; NHL, non-Hodgkin's lymphoma; T2D,type 2 diabetes; NA, not a pplicable.

Note. Low, moderate and high were defined as follow:

Low was defined as ethanol intake of ＞0 g/day and ≤14.9 g/day (about ＞0 drink/day and ＜1 drink/day).

Moderate was defined as ethanol intake of 15–29.9 g/day (about 1-2.5 drinks/day).

High was defined as ethanol intake of ≥30 g/day (about ＞2.5drinks/day).

1 Islami, F. *et al.* Alcohol drinking and esophageal squamous cell carcinoma with focus on light‐drinkers and never‐smokers: A systematic review and meta‐analysis. *International journal of cancer* **129**, 2473-2484 (2011).

2 Friberg, E., Orsini, N., Mantzoros, C. S. & Wolk, A. Alcohol intake and endometrial cancer risk: a meta-analysis of prospective studies. *British Journal of Cancer* **103**, 127-131, doi:10.1038/sj.bjc.6605698 (2010).

3 Sun, Q. *et al.* Alcohol consumption and the risk of endometrial cancer: a meta-analysis. *Asia Pacific Journal of Clinical Nutrition* **20**, 125-133 (2011).

4 Larsson, S. C., Drca, N. & Wolk, A. Alcohol consumption and risk of atrial fibrillation: a prospective study and dose-response meta-analysis. *Journal of the American College of Cardiology* **64**, 281-289 (2014).

5 Jung, M.-H. *et al.* The effect of alcohol dose on the development of hypertension in Asian and Western men: systematic review and meta-analysis. *The Korean journal of internal medicine* **35**, 906 (2020).

6 Padilla, H., Gaziano, J. M. & Djoussé, L. Alcohol consumption and risk of heart failure: a meta-analysis. *The Physician and sportsmedicine* **38**, 84-89 (2010).

7 Larsson, S. C., Orsini, N. & Wolk, A. Alcohol consumption and risk of heart failure: a dose–response meta‐analysis of prospective studies. *European journal of heart failure* **17**, 367-373 (2015).

8 Liu, P.-M. *et al.* Alcohol consumption and coronary heart disease in Eastern Asian men: a meta-analysis of prospective cohort studies. *Zhonghua xin xue guan bing za zhi* **38**, 1038-1044 (2010).

9 Yang, Y. *et al.* Alcohol consumption and risk of coronary artery disease: A dose-response meta-analysis of prospective studies. *Nutrition* **32**, 637-644 (2016).

10 Yoon, S.-J. *et al.* The protective effect of alcohol consumption on the incidence of cardiovascular diseases: is it real? A systematic review and meta-analysis of studies conducted in community settings. *BMC public health* **20**, 1-9 (2020).

11 Ronksley, P. E., Brien, S. E., Turner, B. J., Mukamal, K. J. & Ghali, W. A. Association of alcohol consumption with selected cardiovascular disease outcomes: a systematic review and meta-analysis. *Bmj* **342**, d671 (2011).

12 Zhang, C. *et al.* Alcohol intake and risk of stroke: a dose–response meta-analysis of prospective studies. *International journal of cardiology* **174**, 669-677 (2014).

13 Jiménez-Jiménez, F. J., Alonso-Navarro, H., García-Martín, E. & Agúndez, J. A. Alcohol consumption and risk for Parkinson’s disease: a systematic review and meta-analysis. *Journal of neurology* **266**, 1821-1834 (2019).

14 Jayasekara, H., English, D. R., Room, R. & MacInnis, R. J. Alcohol consumption over time and risk of death: a systematic review and meta-analysis. *Am J Epidemiol* **179**, 1049-1059, doi:10.1093/aje/kwu028 (2014).

15 Wang, C. *et al.* Effect of drinking on all-cause mortality in women compared with men: a meta-analysis. *Journal of Women's Health* **23**, 373-381 (2014).

16 Scd, S. C. Alcohol Dosing and Total Mortality in Men and Women. *Arch Int Med* **166**, 2437-2445 (2006).

17 Baliunas, D. O. *et al.* Alcohol as a risk factor for type 2 diabetes: a systematic review and meta-analysis. *Diabetes care* **32**, 2123-2132 (2009).

18 Han, M. The Dose-Response Relationship between Alcohol Consumption and the Risk of Type 2 Diabetes among Asian Men: A Systematic Review and Meta-Analysis of Prospective Cohort Studies. *Journal of Diabetes Research* **2020**, doi:10.1155/2020/1032049 (2020).

19 Koppes, L. L., Dekker, J. M., Hendriks, H. F., Bouter, L. M. & Heine, R. J. Moderate alcohol consumption lowers the risk of type 2 diabetes: a meta-analysis of prospective observational studies. *Diabetes care* **28**, 719-725 (2005).

20 Huang, J., Wang, X. & Zhang, Y. Specific types of alcoholic beverage consumption and risk of type 2 diabetes: A systematic review and meta‐analysis. *Journal of diabetes investigation* **8**, 56-68 (2017).

21 Pereira, P. P. d. S., Mata, F. A. F. D., Figueiredo, A. C. M. G., Silva, R. B. & Pereira, M. G. Maternal exposure to alcohol and low birthweight: a systematic review and meta-analysis. *Revista Brasileira de Ginecologia e Obstetrícia* **41**, 333-347 (2019).
