# Supplementary material for: Alcohol and Health Outcomes: An Umbrella Review of Meta-Analyses Base on Prospective Cohort Studies
Source: Front Public Health. 2022 May 4;10:859947. doi: 10.3389/fpubh.2022.859947 (PMC9115901; doi:10.3389/fpubh.2022.859947)
Supplement: Supplementary file 2 [file Table_2.docx]

**ESM Table 2.** General characteristics and main findings of the **140 unique meta-analyses retained**

| Health outcome | Author, year | Studies | subjects | Cases | Alcohol intake (g/day) vs none: | Effect size | | Heterogeneity | | Small-study effect |
| --- | --- | --- | --- | --- | --- | --- | --- | --- | --- | --- |
|  |  |  |  |  |  | Relative risk and 95% CIs | P-value | I^2^ | P-value |  |
| **Cancers** |  |  |  |  |  |  |  |  |  |  |
| **Risk** |  |  |  |  |  |  |  |  |  |  |
| Liver cancer^1^ | Turati et al, 2014 | 6 | 2351580 | 2309 | Low | **0.73(0.54-0.98)** | **0.036** | 87.30% | 0.000 | 0.642 |
| Liver cancer^1^ | Turati et al, 2014 | 6 | 1543010 | 1546 | Moderate | 0.95(0.73-1.24) | 0.725 | 73.80% | 0.002 | 0.784 |
| Liver cancer^1^ | Turati et al, 2014 | 7 | 1256838 | 1879 | High | 1.18(0.85-1.63) | 0.324 | 90.40% | 0.000 | 0.155 |
| Colon cancer^2^ | Moskal et al, 2006 | 10 | 919422 | 2081 | Low | 0.99(0.83-1.18) | 0.916 | 64.40% | 0.001 | 0.343 |
| Colon cancer^2^ | Moskal et al, 2006 | 9 | 798510 | 1378 | Moderate | **1.27(1.09-1.49)** | **0.003** | 40.20% | 0.099 | 0.722 |
| Colon cancer^2^ | Moskal et al, 2006 | 7 | 240421 | 1567 | High | 1.35(0.94-1.93) | 0.101 | 76.10% | 0.000 | 0.616 |
| Rectum cancer^2^ | Moskal et al, 2006 | 7 | 307001 | 505 | Low | 1.28(0.96-1.70) | 0.091 | 51.30% | 0.055 | 0.573 |
| Rectum cancer^2^ | Moskal et al, 2006 | 7 | 618203 | 568 | Moderate | **1.44(1.06-1.95)** | **0.020** | 66.60% | 0.006 | 0.279 |
| Rectum cancer^2^ | Moskal et al, 2006 | 5 | 96251 | 439 | High | **1.53(1.22-2.18)** | **0.001** | 0.00% | 0.628 | 0.551 |
| Colorectal cancer^2^ | Moskal et al, 2006 | 3 | 543206 | 707 | Low | 1.03(0.83-1.27) | 0.788 | 41.80% | 0.161 | 0.003 |
| Colorectal cancer^2^ | Moskal et al, 2006 | 5 | 595466 | 767 | Moderate | **1.23(1.02-1.48)** | **0.029** | 0.00% | 0.429 | 0.912 |
| Pancreatic cancer^3^ | Wang et al, 2016 | 10 | 1599723 | 8466 | Low | 0.87(0.75-1.01) | 0.060 | 87.60% | 0.000 | 0.532 |

**(*continued*)**

| Health outcome | Author, year | Studies | subjects | Cases | Alcohol intake (g/day) vs none: | Effect size | | Heterogeneity | | Small-study effect |
| --- | --- | --- | --- | --- | --- | --- | --- | --- | --- | --- |
|  |  |  |  |  |  | Relative risk and 95% CIs | P-value | I^2^ | P-value |  |
| Pancreatic cancer^3^ | Wang et al, 2016 | 10 | 1674699 | 5803 | Moderate | 0.89(0.68-1.15) | 0.355 | 89.10% | 0.000 | 0.315 |
| Pancreatic cancer^3^ | Wang et al, 2016 | 9 | 1423783 | 5778 | High | 1.16(0.71-1.63) | 0.738 | 95.20% | 0.000 | 0.609 |
| Gastric cancer^4^ | He et al, 2017 | 11 | 2905518 | 12503 | Low | 0.90(0.81-1.01) | 0.062 | 51.3% | 0.011 | 0.884 |
| Gastric cancer^4^ | He et al, 2017 | 13 | 1665549 | 6944 | Moderate | 0.98(0.89-1.09) | 0.735 | 30.40% | 0.141 | 0.901 |
| Gastric cancer^4^ | He et al, 2017 | 16 | 1691000 | 9108 | High | **1.15(1.01-1.31)** | **0.036** | 62.70% | 0.000 | 0.364 |
| Esophageal cancer^5^ | Li et al,2014 | 4 | 982373 | 1560 | Low | **1.47(1.06-2.04)** | **0.022** | 68.20% | 0.024 | 0.574 |
| Esophageal cancer^5^ | Li et al, 2014 | 3 | 342800 | 752 | Moderate | **1.35(1.08-1.69)** | **0.009** | 0.00% | 0.558 | 0.143 |
| Esophageal cancer^5^ | Li et al,2014 | 4 | 691695 | 1257 | High | **2.95(1.52-5.73)** | **0.001** | 94.70% | 0.000 | 0.697 |
| Ovarian cancer^6^ | Huang et al, 2015 | 4 | 1372111 | 4440 | Low | 0.99(0.90-1.09) | 0.841 | 35.20% | 0.187 | 0.326 |
| Ovarian cancer^6^ | Huang et al, 2015 | 4 | 640779 | 2374 | Moderate | 1.00(0.95-1.06) | 0.924 | 0.00% | 0.498 | 0.384 |
| Ovarian cancer^6^ | Huang et al, 2015 | 3 | 445508 | 2008 | High | 0.97(0.89-1.07) | 0590 | 0.00% | 0808 | 0.138 |
| Breast cancer^7^ | Sun et al, 2020 | 18 | 3702738 | 32776 | Low | **1.10(1.00-1.20)** | **0.001** | 91.70% | 0.000 | 0.410 |
| Breast cancer^7^ | Sun et al, 2020 | 13 | 1326526 | 13932 | Moderate | **1.30(1.13-1.49)** | **0.000** | 88.10% | 0.000 | 0.881 |
| Breast cancer^7^ | Sun et al, 2020 | 8 | 1015966 | 10081 | High | **1.47(1.33-1.61)** | **0.000** | 68.30% | 0.002 | 0.887 |

**(*continued*)**

| Health outcome | Author, year | Studies | subjects | Cases | Alcohol intake (g/day) vs none: | Effect size | | Heterogeneity | | Small-study effect |
| --- | --- | --- | --- | --- | --- | --- | --- | --- | --- | --- |
|  |  |  |  |  |  | Relative risk and 95% CIs | P-value | I^2^ | P-value |  |
| Endometrial cancer^8^ | Zhou et al, 2016 | 6 | 1482015 | 6783 | Low | **0.92(0.85-0.99)** | **0.024** | 49.70% | 0.0036 | 0.014 |
| Endometrial cancer^8^ | Zhou et al, 2016 | 5 | 703044 | 3286 | Moderate | 0.91(0.70-1.18) | 0.468 | 85.20% | 0.000 | 0.101 |
| Endometrial cancer^8^ | Zhou et al, 2016 | 4 | 515199 | 2478 | High | 0.99(0.73-1.34) | 0.946 | 84.10% | 0.000 | 0.032 |
| Thyroid cancer^9^ | Hong et al, 2017 | 5 | 1569560 | 1213 | Low | 0.91(0.82-1.01) | 0.078 | 0.00% | 0.873 | 0.509 |
| Thyroid cancer^9^ | Hong et al, 2017 | 5 | 836059 | 577 | Moderate | **0.70(0.58-0.85)** | **0.000** | 0.00% | 0.916 | 0.053 |
| Thyroid cancer^9^ | Hong et al, 2017 | 2 | 547947 | 262 | High | **0.55(0.38-0.78)** | **0.001** | 0.00% | 0.765 | NA |
| Renal cell carcinoma^10^ | Xu et al, 2015 | 8 | 1849631 | 3318 | Low | **0.86(0.79-0.93)** | **0.000** | 18.60% | 0.288 | 0.988 |
| Renal cell carcinoma^10^ | Xu et al, 2015 | 4 | 1057344 | 1797 | Moderate | **0.71(0.63-0.80)** | **0.000** | 0.00% | 0.988 | 0.590 |
| Renal cell carcinoma^10^ | Xu et al, 2015 | 3 | 512384 | 930 | High | **0.56(0.43-0.73)** | **0.000** | 0.00% | 0.593 | 0.723 |
| Basal cell carcinoma^11^ | Yen et al, 2017 | 3 | 82352 | 2491 | Low | **1.23(1.11-1.37)** | **0.000** | 0.00% | 0.518 | 0.735 |
| Basal cell carcinoma^11^ | Yen et al, 2017 | 3 | 38331 | 1583 | Moderate | **1.37(1.18-1.59)** | **0.000** | 0.00% | 0.604 | 0.713 |
| Basal cell carcinoma^11^ | Yen et al, 2017 | 3 | 28408 | 976 | High | 1.26(0.82-1.93) | 0.291 | 59.0% | 0.278 | NA |
| Cutaneous squamous cell carcinoma^11^ | Yen et al, 2017 | 3 | 199000 | 2226 | Low | **1.15(1.04-1.27)** | **0.005** | 0.00% | 0.602 | 0.674 |
| Cutaneous squamous cell carcinoma^11^ | Yen et al, 2017 | 3 | 125302 | 1579 | Moderate | **1.28(1.16-1.41)** | **0.000** | 0.00% | 0.429 | 0.987 |
| Cutaneous squamous cell carcinoma^11^ | Yen et al, 2017 | 2 | 109172 | 1314 | High | **1.39(1.22-1.58)** | **0.000** | 0.00% | 0.464 | NA |

**(*continued*)**

| Health outcome | Author, year | Studies | subjects | Cases | Alcohol intake (g/day) vs none: | Effect size | | Heterogeneity | | Small-study effect |
| --- | --- | --- | --- | --- | --- | --- | --- | --- | --- | --- |
|  |  |  |  |  |  | Relative risk and 95% CIs | P-value | I^2^ | P-value |  |
| **Mortality** |  |  |  |  |  |  |  |  |  |  |
| Colorectal cancer mortality^12^ | Kim et al, 2019 | 6 | 13124 | 3194 | Low | **0.77(0.67-0.88)** | **0.000** | 24.80% | 0.248 | 0.467 |
| Colorectal cancer mortality^12^ | Kim et al, 2019 | 9 | 12456 | 2981 | Moderate | **0.86(0.74-0.99)** | **0.034** | 45.70% | 0.087 | 0.934 |
| Colorectal cancer mortality^12^ | Kim et al, 2019 | 4 | 6610 | 944 | High | 0.96(0.75-1.24) | 0.755 | 60.20% | 0.040 | 0.361 |
| Esophageal cancer mortality^13^ | Islami et al, 2011 | 2 | 157869 | 85 | Low | 1.34(0.81-2.22) | 0.251 | 0.00% | 0.775 | NA |
| Esophageal cancer mortality^13^ | Islami et al, 2011 | 2 | 178086 | 84 | Moderate | **1.69(1.01-2.81)** | **0.044** | 0.00% | 0.612 | NA |
| Esophageal cancer mortality^13^ | Islami et al, 2011 | 3 | 184183 | 137 | High | **4.67(3.51-6.21)** | **0.000** | 0.00% | 0.784 | NA |
| All cancers mortality^14^ | Jin et al,2012 | 13 | 2210427 | 18778 | Low | **0.89(0.84-0.95)** | **0.000** | 43.30% | 0.034 | 0.525 |
| All cancers mortality^14^ | Jin et al,2012 | 11 | 1110810 | 12375 | Moderate | 1.09(0.95-1.26) | 0.218 | 74.40% | 0.000 | 0.643 |
| All cancers mortality^14^ | Jin et al,2012 | 10 | 2548292 | 18799 | High | **1.36(1.15-1.62)** | **0.000** | 93.00% | 0.000 | 0.063 |
| **Hematological malignancies** |  |  |  |  |  |  |  |  |  |  |
| **Risk** |  |  |  |  |  |  |  |  |  |  |
| NHL^15^ | Psaltopoulou et al, 2018 | 3 | 197879 | 1811 | Low | 0.88(0.70-1.12) | 0.300 | 46.10% | 0.135 | 0.703 |
| NHL^15^ | Psaltopoulou et al, 2018 | 2 | 127357 | 1007 | Moderate | 0.93(0.80-1.07) | 0.296 | 0.00% | 0.519 | NA |
| NHL^15^ | Psaltopoulou et al, 2018 | 2 | 181168 | 1051 | High | **0.82(0.70-0.96)** | **0.013** | 0.00% | 0.386 | NA |

**(*continued*)**

| Health outcome | Author, year | Studies | subjects | Cases | Alcohol intake (g/day) vs none: | Effect size | | Heterogeneity | | Small-study effect |
| --- | --- | --- | --- | --- | --- | --- | --- | --- | --- | --- |
|  |  |  |  |  |  | Relative risk and 95% CIs | P-value | I^2^ | P-value |  |
| **Circulatory system disease** |  |  |  |  |  |  |  |  |  |  |
| **Risk** |  |  |  |  |  |  |  |  |  |  |
| Atrial fibrillation^16^ | Gallagher et al, 2017 | 7 | 119651 | 2070 | Low | 0.70(0.48-1.02) | 0.063 | 93.80% | 0.000 | 0.637 |
| Atrial fibrillation^16^ | Gallagher et al, 2017 | 6 | 129751 | 716 | Moderate | 0.95(0.76-1.19) | 0.659 | 25.40% | 0.244 | 0.735 |
| Atrial fibrillation^16^ | Gallagher et al, 2017 | 6 | 99923 | 867 | High | 1.06(0.85-1.33) | 0.600 | 92.20% | 0.000 | 0.807 |
| Abdominal aortic aneurysm^17^ | Spencer et al, 2017 | 6 | 277581 | 1494 | Low | 0.99(0.80-1.22) | 0.902 | 66.30% | 0.003 | 0.976 |
| Abdominal aortic aneurysm^17^ | Spencer et al, 2017 | 6 | 129751 | 716 | Moderate | 0.95(0.76-1.19) | 0.659 | 25.40% | 0.244 | 0.735 |
| Abdominal aortic aneurysm^17^ | Spencer et al, 2017 | 6 | 99923 | 867 | High | 1.15(0.95-1.39) | 0.147 | 0.00% | 0.664 | 0.223 |
| Hypertension^18^ | Briasoulis et al, 2012 | 8 | 269131 | 31026 | Low | **0.90(0.84-0.97)** | **0.006** | 60.90% | 0.002 | 0.206 |
| Hypertension^18^ | Briasoulis et al, 2012 | 9 | 156225 | 12574 | Moderate | 0.99(0.89-1.10) | 0.822 | 42.80% | 0.082 | 0.164 |
| Hypertension^18^ | Briasoulis et al, 2012 | 9 | 91261 | 8420 | High | **1.63(1.29-2.06)** | **0.000** | 90.70% | 0.000 | 0.096 |
| CVD in patients with hypertension^19^ | Huang et al, 2014 | 7 | 52082 | 5256 | Low | **0.83(0.76-0.90)** | **0.000** | 69.90% | 0.000 | 0.532 |
| CVD in patients with hypertension^19^ | Huang et al, 2014 | 7 | 26315 | 2049 | Moderate | **0.63(0.56-0.72)** | **0.000** | 43.60% | 0.100 | 0.211 |
| CVD in patients with hypertension^19^ | Huang et al, 2014 | 5 | 14763 | 1312 | High | **0.68(0.56-0.84)** | **0.000** | 68.90% | 0.005 | 0.662 |
| Venous thromboembolism^20^ | Chen et al, 2020 | 2 | 52986 | 860 | Low | **0.60(0.50-0.72)** | **0.000** | 41.70% | 0.162 | NA |

**(*continued*)**

| Health outcome | Author, year | Studies | subjects | Cases | Alcohol intake (g/day) vs none: | Effect size | | Heterogeneity | | Small-study effect |
| --- | --- | --- | --- | --- | --- | --- | --- | --- | --- | --- |
|  |  |  |  |  |  | Relative risk and 95% CIs | P-value | I^2^ | P-value |  |
| Venous thromboembolism^20^ | Chen et al, 2020 | 3 | 16451 | 337 | Moderate | **0.64(0.46-0.91)** | **0.012** | 64.10% | 0.062 | 0.156 |
| Heart failure^21^ | Larsson et al, 2018 | 6 | 221845 | 6984 | Low | **0.57(0.45-0.72)** | **0.000** | 96.20% | 0.000 | 0.696 |
| Heart failure^21^ | Larsson et al, 2018 | 7 | 102642 | 5207 | Moderate | **0.63(0.50-0.79)** | **0.000** | 81.30% | 0.000 | 0.622 |
| Heart failure^21^ | Larsson et al, 2018 | 8 | 64804 | 3596 | High | **0.64(0.48-0.84)** | **0.000** | 89.90% | 0.000 | 0.226 |
| CHD^22^ | Ronksley et al, 2011 | 13 | 106021 | 4816 | Low | **0.74(0.62-0.88)** | **0.001** | 84.90% | 0.000 | 0.909 |
| CHD^22^ | Ronksley et al, 2011 | 11 | 58635 | 2197 | Moderate | **0.71(0.58-0.86)** | **0.001** | 68.40% | 0.000 | 0.264 |
| CHD^22^ | Ronksley et al, 2011 | 11 | 87882 | 2743 | High | **0.68(0.56-0.82)** | **0.000** | 74.30% | 0.000 | 0.704 |
| Myocardial infarction^23^ | Yang et al, 2016 | 3 | 53029 | 1064 | Low | 0.89(0.73-1.09) | 0.704 | 0.00% | 0.662 | NA |
| Myocardial infarction^23^ | Yang et al, 2016 | 3 | 50581 | 799 | High | **0.57(0.44-0.75)** | **0.000** | 0.00% | 0.903 | NA |
| **Mortality** |  |  |  |  |  |  |  |  |  |  |
| CHD mortality ^24^ | Zhao et al, 2017 | 26 | 1019402 | 28582 | Low | **0.76(0.67-0.87)** | **0.000** | 94.60% | 0.000 | 0.893 |
| CHD mortality ^24^ | Zhao et al, 2017 | 30 | 712336 | 24230 | Moderate | **0.76(0.68-0.85)** | **0.000** | 81.70% | 0.000 | 0.932 |
| CHD mortality ^24^ | Zhao et al, 2017 | 30 | 716986 | 23519 | High | **0.81(0.73-0.90)** | **0.000** | 82.40% | 0.000 | 0.884 |
| CVD mortality ^22^ | Ronksley et al, 2011 | 9 | 334514 | 5720 | Low | **0.78(0.70-0.87)** | **0.000** | 74.50% | 0.000 | 0.586 |
| CVD mortality ^22^ | Ronksley et al, 2011 | 8 | 170691 | 4077 | Moderate | **0.79(0.70-0.90)** | **0.000** | 45.60% | 0.075 | 0.054 |

**(*continued*)**

| Health outcome | Author, year | Studies | subjects | Cases | Alcohol intake (g/day) vs none: | Effect size | | Heterogeneity | | Small-study effect |
| --- | --- | --- | --- | --- | --- | --- | --- | --- | --- | --- |
|  |  |  |  |  |  | Relative risk and 95% CIs | P-value | I^2^ | P-value |  |
| CVD mortality ^22^ | Ronksley et al, 2011 | 7 | 214341 | 7787 | High | 0.98(0.84-1.16) | 0.833 | 85.00% | 0.000 | 0.078 |
| CHD mortality in patients with T2D^25^ | Koppes et al, 2006 | 4 | 2869 | 300 | Low | **0.69(0.53-0.91)** | **0.007** | 20.40% | 0.280 | 0.941 |
| CHD mortality in patients with T2D^25^ | Koppes et al, 2006 | 4 | 1807 | 149 | Moderate | **0.32(0.16-0.62)** | **0.001** | 42.30% | 0.188 | 0.596 |
| ACM in patients with hypertension^19^ | Huang et al, 2014 | 4 | 880101 | 5022 | Low | **0.81(0.76-0.85)** | **0.000** | 10.90% | 0.346 | 0.668 |
| ACM in patients with hypertension^19^ | Huang et al, 2014 | 4 | 26315 | 2049 | Moderate | **0.81(0.75-0.88)** | **0.000** | 0.00% | 0.717 | 0.734 |
| ACM in patients with hypertension^19^ | Huang et al, 2014 | 4 | 1005 | 493 | High | 0.98(0.87-1.10) | 0.741 | 0.00% | 0.724 | 0.586 |
| **Nervous system outcomes** |  |  |  |  |  |  |  |  |  |  |
| **Risk** |  |  |  |  |  |  |  |  |  |  |
| Total stroke^26^ | Larsson et al, 2016 | 13 | 361053 | 11725 | Low | **0.68(0.58-0.80)** | **0.000** | 92.30% | 0.000 | 0.685 |
| Total stroke^26^ | Larsson et al, 2016 | 13 | 213057 | 8121 | Moderate | 0.84(0.68-1.04) | 0.109 | 90.40% | 0.000 | 0.951 |
| Total stroke^26^ | Larsson et al, 2016 | 10 | 210876 | 9411 | High | 1.07(0.97-1.18) | 0.179 | 64.30% | 0.000 | 0.586 |
| Hemorrhagic stroke^26^ | Larsson et al, 2016 | 8 | 343081 | 2360 | Low | **0.73(0.63-0.85)** | **0.000** | 51.00% | 0.021 | 0.724 |
| Hemorrhagic stroke^26^ | Larsson et al, 2016 | 7 | 208493 | 2143 | Moderate | 0.99(0.85-1.14) | 0.843 | 32.40% | 0.181 | 0.267 |
| Hemorrhagic stroke^26^ | Larsson et al, 2016 | 6 | 142285 | 2306 | High | **1.34(1.16-1.55)** | **0.000** | 38.10% | 0.105 | 0.741 |
| Intracerebral hemorrhage^26^ | Larsson et al, 2016 | 4 | 149062 | 892 | Low | **0.77(0.64-0.94)** | **0.008** | 8.80% | 0.360 | 0.588 |

**(*continued*)**

| Health outcome | Author, year | Studies | subjects | Cases | Alcohol intake (g/day) vs none: | Effect size | | Heterogeneity | | Small-study effect |
| --- | --- | --- | --- | --- | --- | --- | --- | --- | --- | --- |
|  |  |  |  |  |  | Relative risk and 95% CIs | P-value | I^2^ | P-value |  |
| Intracerebral hemorrhage^26^ | Larsson et al, 2016 | 3 | 38802 | 935 | Moderate | 0.93(0.77-1.13) | 0.485 | 0.00% | 0.904 | 0.417 |
| Intracerebral hemorrhage^26^ | Larsson et al, 2016 | 4 | 52467 | 752 | High | 1.25(1.00-1.57) | 0.055 | 18.90% | 0.286 | 0.616 |
| Ischemic stroke^26^ | Larsson et al, 2016 | 15 | 455758 | 8711 | Low | **0.73(0.65-0.82)** | **0.000** | 84.10% | 0.000 | 0.867 |
| Ischemic stroke^26^ | Larsson et al, 2016 | 13 | 254799 | 6732 | Moderate | **0.79(0.66-0.95)** | **0.011** | 86.40% | 0.000 | 0.972 |
| Ischemic stroke^26^ | Larsson et al, 2016 | 13 | 199150 | 5383 | High | 1.02(0.91-1.15) | 0.719 | 67.40% | 0.000 | 0.935 |
| Subarachnoid hemorrhage^26^ | Larsson et al, 2016 | 5 | 245139 | 460 | Low | **1.58(1.03-2.44)** | **0.037** | 52.50% | 0.040 | 0.392 |
| Subarachnoid hemorrhage^26^ | Larsson et al, 2016 | 4 | 119061 | 349 | Moderate | 1.54(0.96-2.46) | 0.392 | 31.00% | 0.227 | 0.085 |
| Subarachnoid hemorrhage^26^ | Larsson et al, 2016 | 5 | 124664 | 419 | High | **1.65(1.23-2.21)** | **0.001** | 0.00% | 0.863 | 0.331 |
| Dementia^27^ | Anstey et al, 2009 | 5 | 9483 | 1127 | Low | **0.66(0.59-0.74)** | **0.000** | 12.50% | 0.334 | 0.375 |
| Dementia^27^ | Anstey et al, 2009 | 5 | 6575 | 654 | Moderate | **0.51(0.31-0.84)** | **0.008** | 87.60% | 0.000 | 0.005 |
| Dementia^27^ | Anstey et al, 2009 | 4 | 2745 | 409 | High | 1.08(0.70-1.67) | 0.723 | 73.80% | 0.010 | 0.327 |
| Alzheimer's disease^27^ | Anstey et al, 2009 | 3 | 1183 | 416 | Low | **0.65(0.53-0.81)** | **0.000** | 20.40% | 0.285 | 0.130 |
| Alzheimer's disease^27^ | Anstey et al, 2009 | 2 | 842 | 241 | Moderate | 0.79(0.60-1.05) | 0.107 | 0.00% | 0.517 | NA |
| Alzheimer's disease^27^ | Anstey et al, 2009 | 3 | 849 | 186 | High | 1.07(0.78-1.48) | 0.659 | 0.00% | 0.343 | 0.775 |
| Parkinson's disease^28^ | Zhang et al, 2014 | 5 | 417481 | 2260 | Low | 1.00(0.89-1.81) | 0.962 | 39.00% | 0.139 | 0.920 |

**(*continued*)**

| Health outcome | Author, year | Studies | subjects | Cases | Alcohol intake (g/day) vs none: | Effect size | | Heterogeneity | | Small-study effect |
| --- | --- | --- | --- | --- | --- | --- | --- | --- | --- | --- |
|  |  |  |  |  |  | Relative risk and 95% CIs | P-value | I^2^ | P-value |  |
| Parkinson's disease^28^ | Zhang et al, 2014 | 4 | 161808 | 1065 | Moderate | 1.03(0.83-1.26) | 0.811 | 53.90% | 0.043 | 0.8432 |
| Parkinson's disease^28^ | Zhang et al, 2014 | 5 | 157763 | 1268 | High | 0.87(0.75-1.01) | 0.073 | 9.30% | 0.356 | 0.666 |
| **Mortality** |  |  |  |  |  |  |  |  |  |  |
| Stroke mortality^22^ | Ronksley et al, 2011 | 6 | 249071 | 3183 | Low | **0.80(0.68-0.94)** | **0.006** | 42.00% | 0.111 | 0.339 |
| Stroke mortality^22^ | Ronksley et al, 2011 | 7 | 192485 | 2535 | Moderate | 0.94(0.81-1.08) | 0.384 | 24.00% | 0.246 | 0.831 |
| Stroke mortality^22^ | Ronksley et al, 2011 | 5 | 199547 | 2682 | High | 1.17(0.97-1.32) | 0.117 | 57.70% | 0.021 | 0.328 |
| ACM^29^ | Stockwell et al, 2015 | 40 | 1198224 | 80082 | Low | **0.76(0.71-0.81)** | **0.000** | 94.00% | 0.000 | 0.726 |
| ACM^29^ | Stockwell et al, 2015 | 42 | 927669 | 95635 | Moderate | 0.95(0.87-1.02) | 0.160 | 94.90% | 0.000 | 0.216 |
| ACM^29^ | Stockwell et al, 2015 | 44 | 1181964 | 104278 | High | 1.10(0.98-1.23) | 0.096 | 98.50% | 0.000 | 0.880 |
| **Metabolic outcomes** |  |  |  |  |  |  |  |  |  |  |
| **Risk** |  |  |  |  |  |  |  |  |  |  |
| Metabolic syndrome^30^ | Sun et al, 2013 | 5 | 37500 | 1603 | Low | 0.99(0.76-1.29) | 0.948 | 86.40% | 0.000 | 0.019 |
| Metabolic syndrome^30^ | Sun et al, 2013 | 5 | 13618 | 1004 | Moderate | 1.25(0.75-2.00) | 0.397 | 91.90% | 0.000 | 0.153 |
| Metabolic syndrome^30^ | Sun et al, 2013 | 4 | 9563 | 884 | High | 1.42(0.71-2.81) | 0.319 | 92.40% | 0.000 | 0.180 |
| Type 2 diabetes^31^ | Li et al, 2016 | 18 | 400835 | 16477 | Low | **0.67(0.60-0.75)** | **0.000** | 84.60% | 0.000 | 0.497 |

**(*continued*)**

| Health outcome | Author, year | Studies | subjects | Cases | Alcohol intake (g/day) vs none: | Effect size | | Heterogeneity | | Small-study effect |
| --- | --- | --- | --- | --- | --- | --- | --- | --- | --- | --- |
|  |  |  |  |  |  | Relative risk and 95% CIs | P-value | I^2^ | P-value |  |
| Type 2 diabetes^31^ | Li et al, 2016 | 19 | 264355 | 17109 | Moderate | **0.69(0.60-0.80)** | **0.000** | 81.70% | 0.000 | 0.185 |
| Type 2 diabetes^31^ | Li et al, 2016 | 12 | 61909 | 2478 | High | 1.10(0.93-1.31) | 0.262 | 69.00% | 0.000 | 0.966 |
| **Pregnancy and childhood outcome** |  |  |  |  |  |  |  |  |  |  |
| **Risk** |  |  |  |  |  |  |  |  |  |  |
| Low birth weight^32^ | Mamluk et al, 2017 | 2 | 5654 | 312 | Low | 0.76(0.55-1.04) | 0.084 | 0.00% | 0.418 | NA |
| Preterm birth^32^ | Mamluk et al, 2017 | 2 | 5654 | 316 | Low | 1.12(0.66-1.88) | 0.683 | 46.8% | 0.170 | NA |
| **Ophthalmic outcomes** |  |  |  |  |  |  |  |  |  |  |
| **Risk** |  |  |  |  |  |  |  |  |  |  |
| Age-related cataracts^33^ | Wang et al, 2014 | 5 | 92058 | 5398 | Low | 0.82(0.63-1.07) | 0.144 | 96.20% | 0.000 | 0.250 |
| Age-related cataracts^33^ | Wang et al, 2014 | 4 | 36120 | 2014 | Moderate | 0.99(0.48-2.07) | 0.984 | 98.00% | 0.000 | 0.228 |
| Age-related cataracts^33^ | Wang et al, 2014 | 3 | 36050 | 2160 | High | 0.72(0.41-1.27) | 0.258 | 94.90% | 0.000 | 0.352 |
| Age-related macular degeneration^34^ | Chong et al, 2007 | 2 | 66081 | 635 | Low | 0.87(0.69-1.10) | 0.246 | 58.60% | 0.064 | 0.694 |
| Age-related macular degeneration^34^ | Chong et al, 2007 | 3 | 34470 | 453 | Moderate | **1.26(1.04-1.52)** | **0.016** | 20.70% | 0.283 | 0.603 |
| **Other health outcomes** |  |  |  |  |  |  |  |  |  |  |

**(*continued*)**

| Health outcome | Author, year | Studies | subjects | Cases | Alcohol intake (g/day) vs none:ers) | Effect size | | Heterogeneity | | Small-study effect |
| --- | --- | --- | --- | --- | --- | --- | --- | --- | --- | --- |
|  |  |  |  |  |  | Relative risk and 95% CIs | P-value | I^2^ | P-value |  |
| **Risk** |  |  |  |  |  |  |  |  |  |  |
| Complete suicide^35^ | Amiri et al, 2020 | 3 | 64977 | 271 | Low | 0.69(0.51-1.04) | 0.067 | 23.10% | 0.274 | 0.580 |
| Complete suicide^35^ | Amiri et al, 2020 | 6 | 56983 | 223 | Moderate | 0.72(0.53-1.00) | 0.051 | 7.00% | 0.367 | 0.538 |
| Complete suicide^35^ | Amiri et al, 2020 | 5 | 64511 | 330 | High | **1.51(1.04-2.19)** | **0.031** | 52.10% | 0.041 | 0.402 |
| Hip fracture^36^ | Zhang et al, 2015 | 2 | 12369 | 529 | Low | 0.99(0.69-1.42) | 0.956 | 77.90% | 0.011 | NA |
| Hip fracture^36^ | Zhang et al, 2015 | 2 | 10566 | 447 | High | 1.17(0.94-1.46) | 0.152 | 0.00% | 0.579 | NA |
| Rheumatoid arthritis^37^ | Jin et al, 2014 | 3 | 24435 | 170 | Low | 0.88(0.63-1.24) | 0.474 | 0.00% | 0.412 | 0.359 |
| Rheumatoid arthritis^37^ | Jin et al, 2014 | 3 | 18129 | 134 | Moderate | 0.87(0.52-1.47) | 0.611 | 0.00% | 0.877 | 0.825 |
| Chronic kidney damage^38^ | Li et al, 2019 | 13 | 80592 | 13944 | Low | **0.88(0.83-0.93)** | **0.020** | 29.50% | 0.149 | 0.690 |
| Chronic kidney damage^38^ | Li et al, 2019 | 9 | 16460 | 2129 | Moderate | **0.76(0.70-0.83)** | **0.035** | 0.00% | 0.554 | 0.770 |
| Chronic kidney damage^38^ | Li et al, 2019 | 14 | 82214 | 12164 | High | **0.80(0.72-0.89)** | **0.000** | 0.00% | 0.401 | 0.074 |
| Frailty^39^ | Kojima et al, 2018 | 3 | 12463 | 2156 | Moderate | **0.71(0.63-0.81)** | **0.000** | 0.000% | 0.632 | 0.054 |

CVD, cardiovascular disease; ACM, all-cause mortality; CHD, coronary heart disease; NHL, non-Hodgkin's lymphoma; NA, not applicable. All significant results are in bold.

Reference

Note. Low, moderate and high were defined as follow:

Low was defined as ethanol intake of ＞0 g/day and ≤14.9 g/day (about ＞0 drink/day and ＜1 drink/day).

Moderate was defined as ethanol intake of 15–29.9 g/day (about 1-2.5 drinks/day).

High was defined as ethanol intake of ≥30 g/day (about ＞2.5drinks/day).

1 Turati, F. *et al.* Alcohol and liver cancer: a systematic review and meta-analysis of prospective studies. *Annals of oncology* **25**, 1526-1535 (2014).

2 Moskal, A., Norat, T., Ferrari, P. & Riboli, E. Alcohol intake and colorectal cancer risk: A dose–response meta‐analysis of published cohort studies. *International journal of cancer* **120**, 664-671 (2007).

3 Wang, Y.-T., Gou, Y.-W., Jin, W.-W., Xiao, M. & Fang, H.-Y. Association between alcohol intake and the risk of pancreatic cancer: a dose–response meta-analysis of cohort studies. *BMC cancer* **16**, 1-11 (2016).

4 He, Z. *et al.* Association between alcohol consumption and the risk of gastric cancer: a meta-analysis of prospective cohort studies. *Oncotarget* **8**, 84459-84472, doi:10.18632/oncotarget.20880 (2017).

5 Li, Y. *et al.* Alcohol drinking and upper aerodigestive tract cancer mortality: a systematic review and meta-analysis. *Oral oncology* **50**, 269-275 (2014).

6 Yan-Hong, H. *et al.* Association between alcohol consumption and the risk of ovarian cancer: a meta-analysis of prospective observational studies. *BMC Public Health* **15**, 223, doi:10.1186/s12889-015-1355-8 (2015).

7 Sun, Q. *et al.* Alcohol consumption by beverage type and risk of breast cancer: a dose-response meta-analysis of prospective cohort studies. *Alcohol and Alcoholism* **55**, 246-253 (2020).

8 Zhou, Q., Guo, P., Li, H. & Chen, X. D. Does alcohol consumption modify the risk of endometrial cancer? A dose–response meta-analysis of prospective studies. *Archives of Gynecology and Obstetrics* **295**, 467-479, doi:10.1007/s00404-016-4263-y (2017).

9 Hong, S.-H., Myung, S.-K., Kim, H. S. & Group, K. M.-A. S. Alcohol intake and risk of thyroid cancer: a meta-analysis of observational studies. *Cancer research and treatment: official journal of Korean Cancer Association* **49**, 534 (2017).

10 Xu, X., Zhu, Y., Zheng, X. & Xie, L. Does beer, wine or liquor consumption correlate with the risk of renal cell carcinoma? A dose-response meta-analysis of prospective cohort studies. *Oncotarget* **6**, 13347 (2015).

11 Yen, H. *et al.* Alcohol intake and risk of nonmelanoma skin cancer: a systematic review and dose–response meta‐analysis. *British Journal of Dermatology* **177**, 696-707 (2017).

12 Kim, Y., Je, Y. & Giovannucci, E. L. Association between Alcohol Consumption and Survival in Colorectal Cancer: A Meta-analysis. *Cancer Epidemiology Biomarkers & Prevention* **28**, 1891-1901, doi:10.1158/1055-9965.Epi-19-0156 (2019).

13 Islami, F. *et al.* Alcohol drinking and esophageal squamous cell carcinoma with focus on light‐drinkers and never‐smokers: A systematic review and meta‐analysis. *International journal of cancer* **129**, 2473-2484 (2011).

14 Jin, M. *et al.* Alcohol drinking and all cancer mortality: a meta-analysis. *Annals of oncology* **24**, 807-816 (2013).

15 Psaltopoulou, T. *et al.* Alcohol consumption and risk of hematological malignancies: a meta‐analysis of prospective studies. *International journal of cancer* **143**, 486-495 (2018).

16 Gallagher, C. *et al.* Alcohol and incident atrial fibrillation–a systematic review and meta-analysis. *International journal of cardiology* **246**, 46-52 (2017).

17 Spencer, S. M., Trower, A. J., Jia, X., Scott, D. J. A. & Greenwood, D. C. Meta-analysis of the association between alcohol consumption and abdominal aortic aneurysm. *British Journal of Surgery* **104**, 1756-1764, doi:10.1002/bjs.10674 (2017).

18 Briasoulis, A., Agarwal, V. & Messerli, F. H. Alcohol consumption and the risk of hypertension in men and women: a systematic review and meta‐analysis. *The Journal of Clinical Hypertension* **14**, 792-798 (2012).

19 Huang, C. *et al.* in *Mayo Clinic Proceedings.* 1201-1210 (Elsevier).

20 Chen, M., Ji, M., Chen, T., Hong, X. & Jia, Y. Alcohol consumption and risk for venous thromboembolism: a meta-analysis of prospective studies. *Frontiers in nutrition* **7**, 32 (2020).

21 Larsson, S. C., Wallin, A. & Wolk, A. Alcohol consumption and risk of heart failure: Meta-analysis of 13 prospective studies. *Clinical Nutrition* **37**, 1247-1251 (2018).

22 Ronksley, P. E., Brien, S. E., Turner, B. J., Mukamal, K. J. & Ghali, W. A. Association of alcohol consumption with selected cardiovascular disease outcomes: a systematic review and meta-analysis. *Bmj* **342**, d671 (2011).

23 Yang, Y. *et al.* Alcohol consumption and risk of coronary artery disease: A dose-response meta-analysis of prospective studies. *Nutrition* **32**, 637-644 (2016).

24 Zhao, J., Stockwell, T., Roemer, A., Naimi, T. & Chikritzhs, T. Alcohol consumption and mortality from coronary heart disease: an updated meta-analysis of cohort studies. *Journal of studies on alcohol and drugs* **78**, 375-386 (2017).

25 Koppes, L. L., Dekker, J. M., Hendriks, H. F., Bouter, L. M. & Heine, R. J. Meta-analysis of the relationship between alcohol consumption and coronary heart disease and mortality in type 2 diabetic patients. *Diabetologia* **49**, 648-652, doi:10.1007/s00125-005-0127-x (2006).

26 Larsson, S. C., Wallin, A., Wolk, A. & Markus, H. S. Differing association of alcohol consumption with different stroke types: a systematic review and meta-analysis. *BMC medicine* **14**, 1-11 (2016).

27 Anstey, K. J., Mack, H. A. & Cherbuin, N. Alcohol Consumption as a Risk Factor for Dementia and Cognitive Decline: Meta-Analysis of Prospective Studies. *American Journal of Geriatric Psychiatry* **17**, 542-555, doi:10.1097/JGP.0b013e3181a2fd07 (2009).

28 Zhang, D., Jiang, H. & Xie, J. Alcohol intake and risk of Parkinson's disease: a meta‐analysis of observational studies. *Movement Disorders* **29**, 819-822 (2014).

29 Stockwell, T. *et al.* Do “moderate” drinkers have reduced mortality risk? A systematic review and meta-analysis of alcohol consumption and all-cause mortality. *Journal of studies on alcohol and drugs* **77**, 185-198 (2016).

30 Sun, K. *et al.* Alcohol consumption and risk of metabolic syndrome: a meta-analysis of prospective studies. *Clinical nutrition* **33**, 596-602 (2014).

31 Li, X.-H., Yu, F.-f., Zhou, Y.-H. & He, J. Association between alcohol consumption and the risk of incident type 2 diabetes: a systematic review and dose-response meta-analysis. *The American journal of clinical nutrition* **103**, 818-829 (2016).

32 Mamluk, L. *et al.* Low alcohol consumption and pregnancy and childhood outcomes: time to change guidelines indicating apparently ‘safe’levels of alcohol during pregnancy? A systematic review and meta-analyses. *BMJ open* **7**, e015410 (2017).

33 Wang, W. & Zhang, X. Alcohol intake and the risk of age-related cataracts: a meta-analysis of prospective cohort studies. *PLoS One* **9**, e107820 (2014).

34 Chong, E. W.-T., Kreis, A. J., Wong, T. Y., Simpson, J. A. & Guymer, R. H. Alcohol consumption and the risk of age-related macular degeneration: a systematic review and meta-analysis. *American journal of ophthalmology* **145**, 707-715. e702 (2008).

35 Amiri, S. & Behnezhad, S. Alcohol use and risk of suicide: a systematic review and Meta-analysis. *Journal of addictive diseases* **38**, 200-213 (2020).

36 Zhang, X., Yu, Z., Yu, M. & Qu, X. Alcohol consumption and hip fracture risk. *Osteoporosis international* **26**, 531-542 (2015).

37 Jin, Z., Xiang, C., Cai, Q., Wei, X. & He, J. Alcohol consumption as a preventive factor for developing rheumatoid arthritis: a dose-response meta-analysis of prospective studies. *Annals of the rheumatic diseases* **73**, 1962-1967 (2014).

38 Li, D. *et al.* Alcohol Drinking and the Risk of Chronic Kidney Damage: A Meta‐Analysis of 15 Prospective Cohort Studies. *Alcoholism: Clinical and Experimental Research* **43**, 1360-1372 (2019).

39 Kojima, G., Liljas, A., Iliffe, S., Jivraj, S. & Walters, K. A systematic review and meta-analysis of prospective associations between alcohol consumption and incident frailty. *Age and Ageing* **47**, 26-34, doi:10.1093/ageing/afx086 (2018).
