# Supplementary material for: Alcohol and Health Outcomes: An Umbrella Review of Meta-Analyses Base on Prospective Cohort Studies
Source: Front Public Health. 2022 May 4;10:859947. doi: 10.3389/fpubh.2022.859947 (PMC9115901; doi:10.3389/fpubh.2022.859947)
Supplement: Supplementary file 3 [file Table_3.docx]

ESM Table 3. Assessments of AMSTAR2 scores.

| Reference | AMSTAR 2 checklist | | | | | | | | | | | | | | | | Overall assessment quality |
| --- | --- | --- | --- | --- | --- | --- | --- | --- | --- | --- | --- | --- | --- | --- | --- | --- | --- |
|  | NO.1 | NO.2 | NO.3 | NO.4 | NO.5 | NO.6 | NO.7 | NO.8 | NO.9 | NO.10 | NO.11 | NO.12 | NO.13 | NO.14 | NO.15 | NO.16 |  |
| Jin et al, 2012 | Y | N | Y | pY | Y | Y | pY | Y | N | N | Y | Y | Y | Y | Y | Y | Critically low |
| Wang et al, 2014 | Y | N | Y | pY | Y | Y | pY | Y | Y | N | Y | Y | Y | Y | Y | Y | Critically low |
| Anstey et al, 2009 | Y | N | Y | pY | Y | Y | pY | pY | N | N | Y | N | Y | N | N | N | Critically low |
| Sun et al, 2013 | Y | N | Y | Y | N | Y | pY | Y | Y | N | Y | Y | Y | Y | Y | Y | Critically low |
| Gallagher et al, 2017 | Y | N | Y | pY | Y | Y | pY | Y | N | N | Y | Y | Y | Y | Y | Y | Critically low |
| Spencer et al, 2017 | Y | Y | Y | pY | Y | Y | pY | Y | Y | N | Y | Y | Y | Y | Y | Y | Critically low |
| Turati et al, 2014 | Y | N | Y | pY | Y | Y | pY | Y | N | N | Y | Y | Y | Y | Y | Y | Critically low |
| Briasoulis et al, 2012 | Y | N | Y | Y | N | Y | pY | pY | N | N | Y | N | Y | Y | Y | N | Critically low |
| Zhao et al, 2017 | Y | N | Y | pY | Y | Y | pY | Y | N | N | Y | Y | Y | Y | Y | Y | Critically low |
| Chong et al, 2007 | Y | N | Y | Y | Y | Y | pY | Y | N | N | Y | Y | Y | Y | Y | Y | Low |
| Moskal et al, 2006 | Y | N | Y | pY | N | N | pY | Y | N | N | Y | N | Y | Y | Y | N | Critically low |
| Kim et al, 2019 | Y | N | Y | pY | Y | Y | pY | Y | Y | N | Y | Y | Y | N | Y | Y | Critically low |
| Chen et al, 2020 | Y | N | Y | pY | Y | Y | pY | Y | Y | N | Y | Y | Y | Y | Y | Y | Critically low |
| Zhang et al, 2015 | Y | N | Y | pY | Y | Y | pY | pY | N | N | Y | Y | Y | Y | Y | Y | Critically low |
| Jin et al, 2014 | Y | N | Y | Y | Y | Y | Y | Y | Y | N | Y | Y | Y | Y | Y | Y | Low |
| Huang et al, 2015 | Y | N | Y | pY | Y | Y | pY | Y | Y | N | Y | Y | Y | Y | Y | Y | Critically low |
| Li et al, 2019 | Y | N | Y | pY | Y | Y | pY | Y | Y | N | Y | Y | Y | Y | Y | Y | Critically low |
| Stockwell et al, 2015 | Y | Y | Y | Y | Y | Y | pY | Y | Y | N | Y | Y | Y | Y | Y | N | Low |
| Sun et al, 2020 | Y | N | Y | pY | Y | Y | pY | Y | Y | N | Y | Y | Y | Y | Y | Y | Critically low |
| Xu et al, 2015 | Y | N | Y | Y | Y | Y | pY | Y | Y | N | Y | Y | Y | Y | Y | Y | Critically low |
| Li et al, 2016 | Y | N | Y | Y | Y | Y | pY | Y | Y | N | Y | Y | Y | Y | Y | Y | Critically low |
| He et al, 2017 | Y | N | Y | pY | Y | Y | pY | Y | Y | N | Y | Y | Y | Y | Y | Y | Critically low |

***(continued)***

| Reference | AMSTAR 2 checklist | | | | | | | | | | | | | | | | Overall assessment quality |
| --- | --- | --- | --- | --- | --- | --- | --- | --- | --- | --- | --- | --- | --- | --- | --- | --- | --- |
|  | NO.1 | NO.2 | NO.3 | NO.4 | NO.5 | NO.6 | NO.7 | NO.8 | NO.9 | NO.10 | NO.11 | NO.12 | NO.13 | NO.14 | NO.15 | NO.16 |  |
| Larsson et al, 2018 | Y | N | Y | pY | Y | Y | pY | Y | N | N | Y | N | Y | N | Y | Y | Critically low |
| Ronksley et al, 2011 | Y | N | Y | Y | Y | Y | pY | Y | Y | N | Y | Y | Y | Y | Y | Y | Critically low |
| Huang et al, 2014 | Y | N | Y | pY | Y | Y | pY | Y | Y | N | Y | Y | Y | Y | Y | Y | Critically low |
| Kojima et al, 2018 | Y | Y | Y | pY | Y | Y | pY | Y | Y | N | Y | N | Y | N | Y | Y | Critically low |
| Psaltopoulou et al, 2018 | Y | N | Y | pY | N | Y | pY | Y | Y | N | Y | Y | Y | N | Y | Y | Critically low |
| Wang et al, 2016 | Y | N | Y | pY | Y | Y | pY | Y | Y | N | Y | N | Y | Y | Y | Y | Critically low |
| Larsson et al, 2016 | Y | N | Y | pY | Y | Y | pY | Y | Y | N | Y | Y | Y | Y | Y | Y | Critically low |
| Zhou et al, 2016 | Y | Y | Y | Y | Y | Y | pY | Y | Y | N | Y | Y | Y | N | Y | Y | Low |
| Amiri et al, 2020 | Y | N | Y | pY | Y | Y | pY | Y | Y | N | Y | Y | Y | N | Y | Y | Critically low |
| Li et al, 2014 | Y | N | Y | pY | Y | Y | pY | Y | Y | N | Y | Y | Y | Y | Y | Y | Critically low |
| Hong et al, 2017 | Y | N | Y | pY | Y | Y | pY | Y | Y | N | Y | N | Y | N | Y | Y | Critically low |
| Yen et al, 2017 | Y | N | Y | Y | Y | Y | pY | Y | Y | N | Y | N | Y | Y | Y | Y | Critically low |
| Islami et al, 2011 | Y | N | Y | pY | N | N | pY | Y | N | N | Y | N | Y | Y | Y | N | Critically low |
| Yang et al, 2016 | Y | N | Y | pY | Y | Y | pY | Y | N | N | Y | N | Y | N | Y | Y | Critically low |
| Zhang et al, 2014 | Y | N | Y | pY | N | N | pY | Y | N | N | Y | Y | Y | Y | Y | Y | Critically low |
| Mamluk et al, 2017 | Y | Y | Y | pY | Y | Y | pY | Y | Y | N | Y | Y | Y | Y | Y | Y | Critically low |
| Koppes et al, 2006 | Y | N | Y | pY | N | N | pY | Y | N | N | N | N | N | N | N | Y | Critically low |

AMSTAR 2 checklist (items in italic are considered critical):

1, PICO description; 2, protocol registered before the commencement of the review; 3, study design included in the review; 4, adequacy of the literature search; 5, two authors study selection; 6, two authors study extraction; 7, list for excluding individual studies; 8, included studies descripted in detail; 9, risk of bias for the single studies that included in the review; 10, source of funding of primary studies; 11, appropriateness of meta-analytical methods; 12, impact of risk of bias of single studies on the results of the meta-analysis; 13, consideration of risk of bias when interpreting the results of the review; 14 explanation and discussion of the heterogeneity observed; 15, assessment of presence and likely impact of publication bias; 16, funding sources and conflict of interest declared.

Abbreviations: Y, yes; PY, partial yes; N, no.

Footnotes:
High: 0–1 non-critical weakness. The systematic review provides an accurate and comprehensive summary of the results of the available studies that address the question of interest.
Moderate: >1 non-critical weakness. The systematic review has more than one weakness, but no critical flaws. It may provide an accurate summary of the results of the available studies that were included in the review.
Low: 1 critical flaw with or without non-critical weaknesses. The review has a critical flaw and may not provide an accurate and comprehensive summary of the available studies that address the question of interest.
Critically low: >1 critical flaw with or without non-critical weaknesses. The review has more than one critical flaw and should not be relied on to provide an accurate and comprehensive summary of the available studies.
