# Supplementary material for: Alcohol and Health Outcomes: An Umbrella Review of Meta-Analyses Base on Prospective Cohort Studies
Source: Front Public Health. 2022 May 4;10:859947. doi: 10.3389/fpubh.2022.859947 (PMC9115901; doi:10.3389/fpubh.2022.859947)
Supplement: Supplementary file 4 [file Table_4.docx]

**ESM Table 4. General characteristics and main findings of 66 health outcomes that did not show statistically significant associations**

| **Health outcomes** | **Reference** | **precision of the estimate** | | **consistency of results** | **no evidence of small-study effects** |
| --- | --- | --- | --- | --- | --- |
|  |  | **＞1000 disease case** | **P＞0.05** | **I^2^ < 50% and Cochran Q test P > .10** | **P>0.1** |
| **21 health outcomes in low alcohol consumption group.** | | | | | |
| **Risk** | | | | | |
| Colon cancer | Moskal et al, 2006 | Yes | Yes | No | Yes |
| Rectum cancer | Moskal et al, 2006 | No | Yes | No | Yes |
| Colorectal cancer | Moskal et al, 2006 | No | Yes | Yes | No |
| Pancreatic cancer | Wang et al, 2016 | Yes | Yes | No | Yes |
| Gastric cancer | He et al, 2017 | Yes | Yes | No | Yes |
| Ovarian cancer | Huang et al, 2015 | Yes | Yes | Yes | Yes |
| Thyroid cancer | Hong et al, 2017 | Yes | Yes | Yes | Yes |
| NHL | Psaltopoulou et al, 2018 | Yes | Yes | Yes | Yes |
| Atrial fibrillation | Gallagher et al, 2017 | Yes | Yes | No | Yes |
| Abdominal aortic aneurysm | Spencer et al, 2017 | Yes | Yes | No | Yes |
| Myocardial infarction | Yang et al, 2016 | Yes | Yes | Yes | NA |
| Parkinson’s disease | Zhang et al, 2014 | Yes | Yes | Yes | Yes |
| Metabolic syndrome | Sun et al, 2013 | Yes | Yes | Yes | NA |
| Low birth weight | Pereira et al, 2019 | No | Yes | Yes | NA |
| Preterm birth | Pereira et al, 2019 | No | Yes | Yes | NA |
| Age-related cataracts | Wang et al, 2014 | Yes | Yes | No | Yes |
| Age-related macular degeneration | Chong et al, 2007 | No | Yes | No | Yes |
| Complete suicide | Amiri et al, 2020 | No | Yes | Yes | Yes |
| Hip fracture | Zhang et al, 2015 | No | Yes | No | NA |
| Rheumatoid arthritis | Jin et al, 2014 | No | Yes | Yes | Yes |
| **Mortality** |  |  |  |  |  |
| Esophageal cancer mortality | Islami et al, 2011 | No | Yes | Yes | NA |
| **22 health outcomes in moderate alcohol consumption group.** | | | | | |
| **Risk** | | | | | |
| Liver cancer | Turati et al, 2014 | Yes | Yes | No | Yes |
| Pancreatic cancer | Wang et al, 2016 | Yes | Yes | No | Yes |
| Gastric cancer | He et al, 2017 | Yes | Yes | Yes | Yes |
| Ovarian cancer | Huang et al, 2015 | Yes | Yes | Yes | Yes |
| Endometrial cancer | Zhou et al, 2016 | Yes | Yes | No | Yes |
| NHL | Psaltopoulou et al, 2018 | Yes | Yes | Yes | NA |
| Atrial fibrillation | Gallagher et al, 2017 | No | Yes | Yes | Yes |
| Abdominal aortic aneurysm | Spencer et al, 2017 | No | Yes | Yes | Yes |
| Hypertension | Briasoulis et al, 2012 | Yes | Yes | No | Yes |

**(*continued*)**

| **Health outcomes** | **Reference** | **precision of the estimate** | | **consistency of results** | **no evidence of small-study effects** |
| --- | --- | --- | --- | --- | --- |
|  |  | **＞1000 disease case** | **P＞0.05** | **I^2^ < 50% and Cochran Q test P > .10** | **P>0.1** |
| Total stroke | Larsson et al, 2016 | Yes | Yes | No | Yes |
| Hemorrhagic stroke | Larsson et al, 2016 | Yes | Yes | Yes | Yes |
| Intracerebral hemorrhage | Larsson et al, 2016 | No | Yes | Yes | Yes |
| Subarachnoid hemorrhage | Larsson et al, 2016 | No | Yes | Yes | No |
| Alzheimer's disease | Anstey et al, 2009 | No | Yes | Yes | NA |
| Parkinson’s disease | Zhang et al, 2014 | Yes | Yes | Yes | Yes |
| Metabolic syndrome | Sun et al, 2013 | Yes | Yes | No | Yes |
| Age-related cataracts | Wang et al, 2014 | Yes | Yes | No | Yes |
| Complete suicide | Amiri et al, 2020 | No | Yes | Yes | Yes |
| Rheumatoid arthritis | Jin et al, 2014 | No | Yes | Yes | Yes |
| Mortality | | | | | |
| All cancers mortality | Jin et al,2012 | Yes | Yes | No | Yes |
| Stroke mortality | Ronksley et al, 2011 | Yes | Yes | Yes | Yes |
| ACM | Stockwell et al, 2015 | Yes | Yes | No | Yes |
| **23 health outcomes in high alcohol consumption group.** | | | | | |
| **Risk** | | | | | |
| Liver cancer | Turati et al, 2014 | Yes | Yes | No | Yes |
| Colon cancer | Moskal et al, 2006 | Yes | Yes | No | Yes |
| Pancreatic cancer | Wang et al, 2016 | Yes | Yes | No | Yes |
| Ovarian cancer | Huang et al, 2015 | Yes | Yes | Yes | Yes |
| Endometrial cancer | Zhou et al, 2016 | Yes | Yes | No | No |
| Basal cell carcinoma | Yen et al, 2017 | No | Yes | No | NA |
| Atrial fibrillation | Gallagher et al, 2017 | No | Yes | No | Yes |
| Abdominal aortic aneurysm | Spencer et al, 2017 | No | Yes | Yes | Yes |
| Total stroke | Larsson et al, 2016 | Yes | Yes | No | Yes |
| Intracerebral hemorrhage | Larsson et al, 2016 | No | Yes | Yes | Yes |
| Ischemic stroke | Larsson et al, 2016 | Yes | Yes | No | Yes |
| Dementia | Anstey et al, 2009 | No | Yes | No | Yes |
| Alzheimer's disease | Anstey et al, 2009 | No | Yes | Yes | Yes |
| Parkinson’s disease | Zhang et al, 2014 | Yes | Yes | Yes | Yes |
| Metabolic syndrome | Sun et al, 2013 | No | Yes | No | Yes |
| Type 2 diabetes | Li et al, 2016 | Yes | Yes | No | Yes |
| Age-related cataracts | Wang et al, 2014 | Yes | Yes | No | Yes |
| Hip fracture | Zhang et al, 2015 | No | Yes | Yes | NA |
| **Mortality** |  |  |  |  |  |
| Colorectal cancer mortality | Kim et al, 2019 | No | Yes | No | Yes |

**(*continued*)**

| **Health outcomes** | **Reference** | **precision of the estimate** | | **consistency of results** | **no evidence of small-study effects** |
| --- | --- | --- | --- | --- | --- |
|  |  | **＞1000 disease case** | **P＞0.05** | **I^2^ < 50% and Cochran Q test P > .10** | **P>0.1** |
| CVD mortality | Ronksley et al, 2011 | Yes | Yes | No | No |
| ACM in patients with hypertension | Huang et al, 2014 | No | Yes | Yes | Yes |
| Stroke mortality | Ronksley et al, 2011 | Yes | Yes | No | Yes |
| ACM | Stockwell et al, 2015 | Yes | Yes | No | Yes |

NHL, non-Hodgkin's lymphoma; ACM, all-cause mortality; CVD, cardiovascular disease; NA, not applicable.
