# Supplementary material for: Alcohol and Health Outcomes: An Umbrella Review of Meta-Analyses Base on Prospective Cohort Studies
Source: Front Public Health. 2022 May 4;10:859947. doi: 10.3389/fpubh.2022.859947 (PMC9115901; doi:10.3389/fpubh.2022.859947)
Supplement: Supplementary file 5 [file Table_5.docx]

**ESM Table 5. The strength of epidemiologic evidence of 29 health outcomes in low alcohol consumption group.**

| **Health outcomes** | **Reference** | **precision of the estimate** | | **consistency of results** | **no evidence of small-study effects** | **Grade** |
| --- | --- | --- | --- | --- | --- | --- |
|  |  | **>1000 disease cases** | **P<0.001** | **I^2^ < 50% and Cochran Q test P > .10** | **P>0.1** |  |
| **24 beneficial health outcomes** | | | | | | |
| **Risk** | | | | | | |
| Liver cancer | Turati et al, 2014 | Yes | No | No | Yes | Weak |
| Endometrial cancer | Zhou et al, 2016 | Yes | No | No | No | Weak |
| Renal cell carcinoma | Xu et al, 2015 | Yes | Yes | Yes | Yes | High |
| Hypertension | Briasoulis et al, 2012 | Yes | No | No | Yes | Weak |
| CVD in patients with hypertension | Huang et al, 2014 | Yes | Yes | No | Yes | Moderate |
| Venous thromboembolism | Chen et al, 2020 | No | No | No | Yes | Weak |
| Heart failure | Larsson et al, 2018 | Yes | Yes | No | Yes | Moderate |
| CHD | Ronksley et al, 2011 | Yes | No | No | Yes | Weak |
| Total stroke | Larsson et al, 2016 | Yes | Yes | No | Yes | Moderate |
| Hemorrhagic stroke | Larsson et al, 2016 | Yes | Yes | No | Yes | Moderate |
| Intracerebral hemorrhage | Larsson et al, 2016 | No | No | Yes | Yes | Weak |
| Ischemic stroke | Larsson et al, 2016 | Yes | Yes | No | Yes | Moderate |

**(*continued*)**

| **Health outcomes** | Reference | **precision of the estimate** | | **consistency of results** | **no evidence of small-study effects** | **Grade** |
| --- | --- | --- | --- | --- | --- | --- |
|  |  | **>1000 disease cases** | **P<0.001** | **I^2^ < 50% and Cochran Q test P > .10** | **P>0.1** |  |
| Dementia | Anstey et al, 2009 | Yes | Yes | Yes | Yes | High |
| Alzheimer's disease | Anstey et al, 2009 | No | Yes | Yes | Yes | Moderate |
| Type 2 diabetes | Li et al, 2016 | Yes | Yes | No | Yes | Moderate |
| Chronic kidney damage | Li et al, 2019 | Yes | No | Yes | Yes | Weak |
| **Mortality** |  |  |  |  |  |  |
| Colorectal cancer mortality | Kim et al, 2019 | Yes | Yes | Yes | Yes | High |
| All cancer mortality | Jin et al,2012 | Yes | Yes | No | Yes | Moderate |
| CHD mortality | Zhao et al, 2017 | Yes | Yes | No | Yes | Moderate |
| CVD mortality | Ronksley et al, 2011 | Yes | No | No | No | Weak |
| CHD mortality in patients with T2D | Koppes et al, 2006 | No | No | Yes | Yes | Weak |
| ACM in patients with hypertension | Huang et al, 2014 | Yes | Yes | Yes | Yes | High |
| Stroke mortality | Ronksley et al, 2011 | Yes | No | Yes | Yes | Weak |
| ACM | Stockwell et al, 2015 | Yes | Yes | No | Yes | Moderate |
| **5 harmful health outcomes** | | | | | | |

**(*continued*)**

| **Health outcomes** | **Reference** | **precision of the estimate** | | **consistency of results** | **no evidence of small-study effects** | **Grade** |
| --- | --- | --- | --- | --- | --- | --- |
|  |  | **>1000 disease cases** | **P<0.001** | **I^2^ < 50% and Cochran Q test P > .10** | **P>0.1** |  |
| Risk |  |  |  |  |  |  |
| Esophageal cancer | Li et al,2014 | Yes | No | No | Yes | Weak |
| Breast cancer | Sun et al, 2020 | Yes | Yes | No | Yes | Moderate |
| Basal cell carcinoma | Yen et al, 2017 | Yes | Yes | Yes | Yes | High |
| Cutaneous squamous cell carcinoma | Yen et al, 2017 | Yes | No | Yes | Yes | Weak |
| Subarachnoid hemorrhage | Larsson et al, 2016 | No | No | No | Yes | Weak |

CVD, cardiovascular disease; ACM, all-cause mortality; CHD, coronary heart disease.

**NOTE. The strength of epidemiologic evidence was rated as follows:**
High, if all criteria were satisfied: precision of the estimate (P < .001 and >1000 disease cases), consistency of results (I2 < 50% and Cochran Q test P > .10), and no evidence of small-study effects (P > .10).
Moderate, if a maximum of 1 criterion was not satisfied and a P < .001 was found.
Weak, in other cases (P < .05).
