# Supplementary material for: Alcohol and Health Outcomes: An Umbrella Review of Meta-Analyses Base on Prospective Cohort Studies
Source: Front Public Health. 2022 May 4;10:859947. doi: 10.3389/fpubh.2022.859947 (PMC9115901; doi:10.3389/fpubh.2022.859947)
Supplement: Supplementary file 7 [file Table_7.docx]

**ESM Table 7. The strength of epidemiologic evidence of 20 health outcomes in high alcohol consumption group.**

| **Health outcomes** | **Reference** | **precision of the estimate** | | **consistency of results** | **no evidence of small-study effects** | **Grade** |
| --- | --- | --- | --- | --- | --- | --- |
|  |  | **>1000 disease cases** | **P<0.001** | **I^2^ < 50% and Cochran Q test P > .10** | **P>0.1** |  |
| **9 beneficial health outcomes** | | | | | | |
| **Risk** | | | | | | |
| Thyroid cancer | Hong et al, 2017 | No | No | Yes | NA | Weak |
| Renal cell carcinoma | Xu et al, 2015 | No | Yes | Yes | Yes | Moderate |
| NHL | Psaltopoulou et al, 2018 | Yes | No | Yes | NA | Weak |
| CVD in patients with hypertension | Huang et al, 2014 | Yes | Yes | No | Yes | Moderate |
| Heart failure | Larsson et al, 2018 | Yes | Yes | No | Yes | Moderate |
| CHD | Ronksley et al, 2011 | Yes | Yes | No | Yes | Moderate |
| Myocardial infarction | Yang et al, 2016 | No | Yes | Yes | NA | Weak |
| Chronic kidney damage | Li et al, 2019 | Yes | Yes | Yes | No | Moderate |
| **Mortality** |  |  |  |  |  |  |
| CHD mortality | Zhao et al, 2017 | Yes | Yes | No | Yes | Moderate |
| **11 harmful health outcomes** | | | | | | |
| **Risk** |  |  |  |  |  |  |

**(*continued*)**

| **Health outcomes** | **Reference** | **precision of the estimate** | | **consistency of results** | **no evidence of small-study effects** | **Grade** |
| --- | --- | --- | --- | --- | --- | --- |
|  |  | **>1000 disease cases** | **P<0.001** | **I^2^ < 50% and Cochran Q test P > .10** | **P>0.1** |  |
| Rectum cancer | Moskal et al, 2006 | No | No | Yes | Yes | Weak |
| Gastric cancer | He et al, 2017 | Yes | No | No | Yes | Weak |
| Esophageal cancer | Li et al,2014 | Yes | No | No | Yes | Weak |
| Breast cancer | Sun et al, 2020 | Yes | Yes | No | Yes | Moderate |
| Cutaneous squamous cell carcinoma | Yen et al, 2017 | Yes | Yes | Yes | NA | Moderate |
| Hypertension | Briasoulis et al, 2012 | Yes | Yes | No | No | Weak |
| Hemorrhagic stroke | Larsson et al, 2016 | Yes | Yes | Yes | Yes | High |
| Subarachnoid hemorrhage | Larsson et al, 2016 | No | No | Yes | Yes | Weak |
| Complete suicide | Amiri et al, 2020 | No | No | No | Yes | Weak |
| **Mortality** | | | | | | |
| Esophageal cancer mortality | Islami et al, 2011 | No | Yes | Yes | NA | Weak |
| All cancer mortality | Jin et al,2012 | Yes | Yes | No | No | Weak |

CVD, cardiovascular disease; CHD, coronary heart disease; NHL, non-Hodgkin's lymphoma; NA, not applicable.

**NOTE. The strength of epidemiologic evidence was rated as follows:**
High, if all criteria were satisfied: precision of the estimate (P < .001 and >1000 disease cases), consistency of results (I2 < 50% and Cochran Q test P > .10), and no evidence of small-study effects (P > .10).
Moderate, if a maximum of 1 criterion was not satisfied and a P < .001 was found.
Weak, in other cases (P < .05).
