# Supplementary material for: Alcohol and Health Outcomes: An Umbrella Review of Meta-Analyses Base on Prospective Cohort Studies
Source: Front Public Health. 2022 May 4;10:859947. doi: 10.3389/fpubh.2022.859947 (PMC9115901; doi:10.3389/fpubh.2022.859947)
Supplement: Supplementary file 8 [file Data_Sheet_1.docx]

# Alcohol and health outcomes: An umbrella review of meta-analyses base on prospective cohort studies.

# Clinical and Experimental Medicine

Lixian Zhong^1†^, Weiwei Chen^1,2†^, Tonghua Wang^3†^, Qiuting Zeng^1^, Leizhen Lai^1^, Junlong Lai^1^, Junqin Lin^1^, Shaohui Tang^1*^

***Correspondence to**: Shaohui Tang, email: [tangshaohui206@jnu.edu.cn](mailto:tangshaohui206@jnu.edu.cn)

Department of Gastroenterology, The First Affiliated Hospital, Jinan University, Guangzhou, Guangdong, 510630, P. R. China.

**Supplementary figure lengends**

**Figure S1.** Forest plot: reacalculated effects estimates of meta-analyses reporting no significant associations of low alcohol consumption with health outcomes. RR, relative risk; CI, confidence interval; NHL, Non-Hodgkin's lymphoma.

**Fig S2.** Forest plot: reacalculated effects estimates of meta-analyses reporting no significant associations of moderate alcohol sonsumption with health outcomes. RR, relative risk; CI, confidence interval; ACM, all-cause mortality.

**Fig S3.** Forest plot: reacalculated effects estimates of meta-analyses reporting no significant associations of high alcohol consumption with health outcomes. RR, relative risk; CI, confidence interval; CVD, cardiovascular disease; ACM, all-cause mortality.


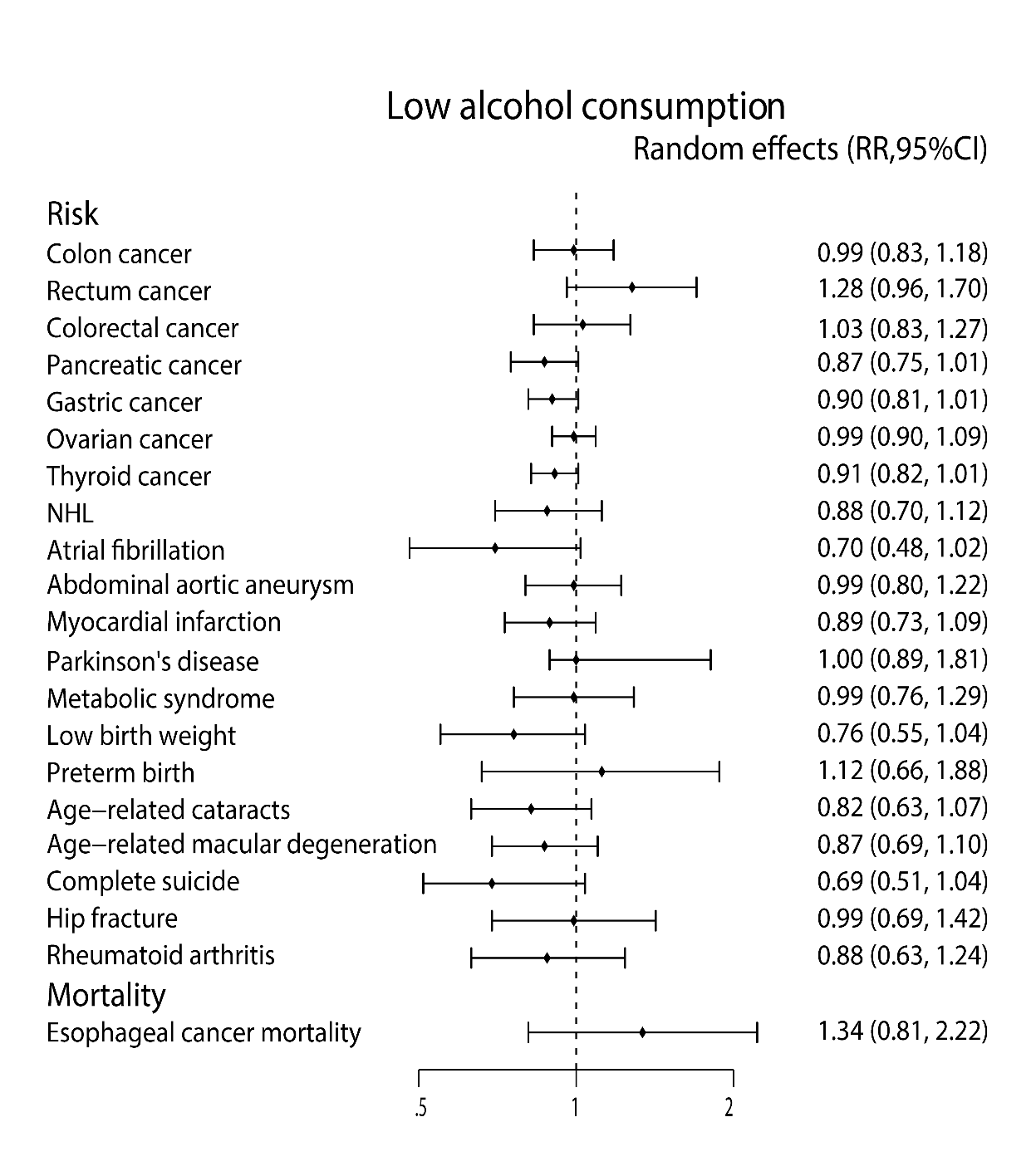


Figure S1


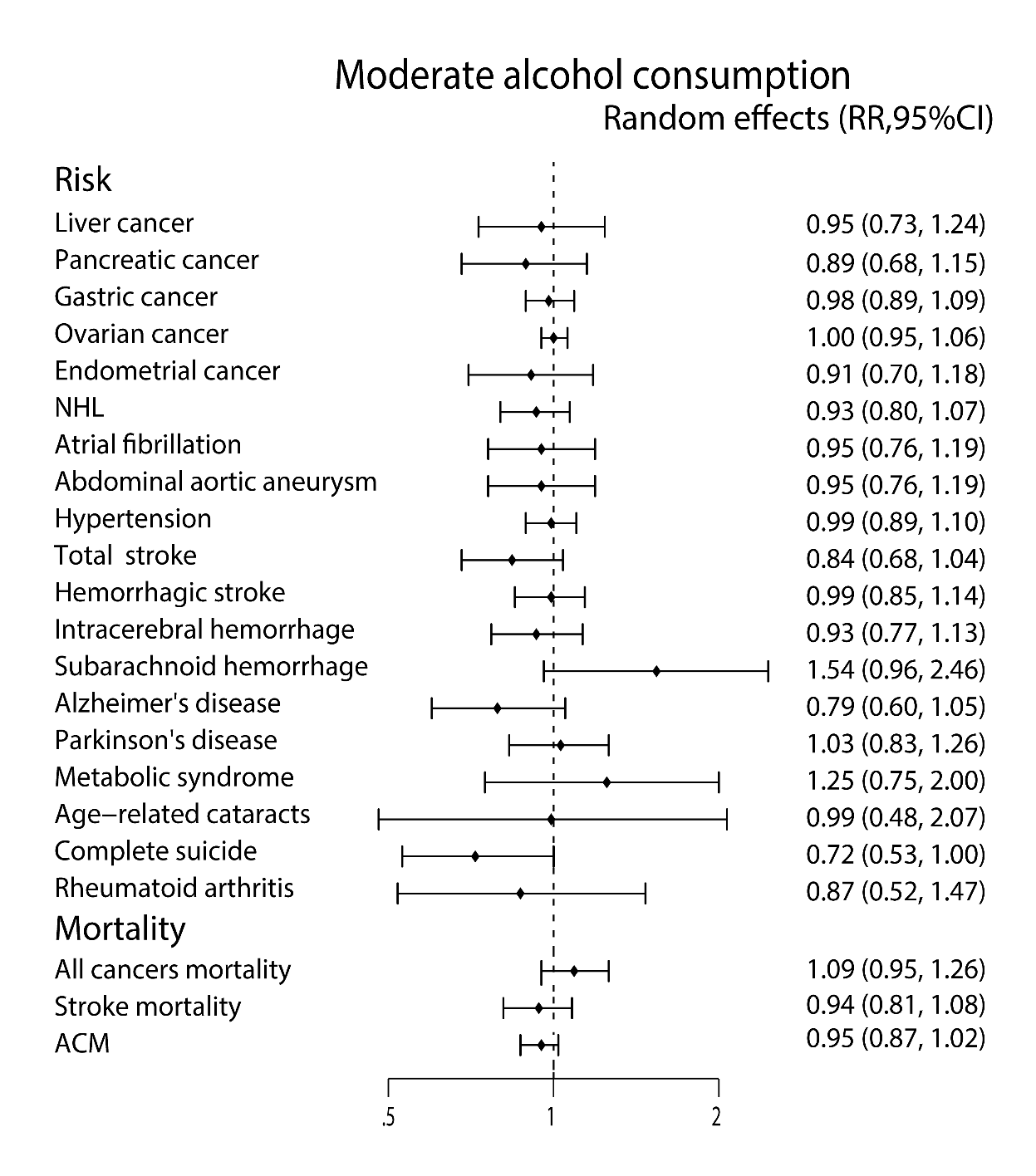


Figure S2


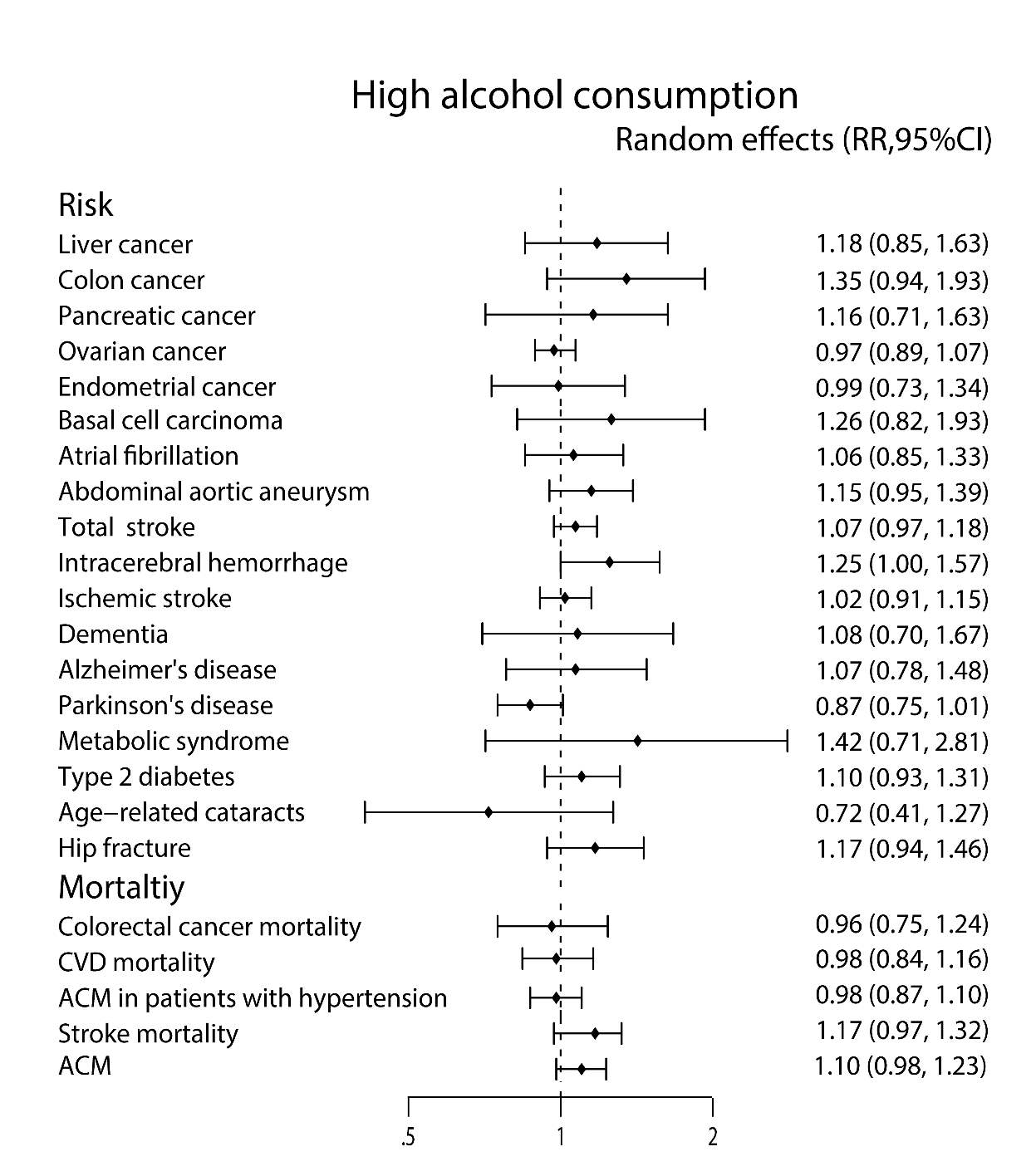


Figure S3
